# The identification of grain size genes by RapMap reveals directional selection during rice domestication

**DOI:** 10.1038/s41467-021-25961-1

**Published:** 2021-09-28

**Authors:** Juncheng Zhang, Dejian Zhang, Yawei Fan, Cuicui Li, Pengkun Xu, Wei Li, Qi Sun, Xiaodong Huang, Chunyu Zhang, Linyue Wu, Huaizhou Yang, Shiyu Wang, Xiaomin Su, Xingxing Li, Yingying Song, Meng-en Wu, Xingming Lian, Yibo Li

**Affiliations:** 1 National Key Laboratory of Crop Genetic Improvement and National Centre of Plant Gene Research (Wuhan), Huazhong Agricultural University, Wuhan, China; 2 Hubei Hongshan Laboratory, Wuhan, China

**Keywords:** Genetic mapping, Agricultural genetics, Natural variation in plants, Plant evolution

## Abstract

Cloning quantitative trait locus (QTL) is time consuming and laborious, which hinders the understanding of natural variation and genetic diversity. Here, we introduce RapMap, a method for rapid multi-QTL mapping by employing F_2_ gradient populations (F_2_GPs) constructed by minor-phenotypic-difference accessions. The co-segregation standard of the single-locus genetic models ensures simultaneous integration of a three-in-one framework in RapMap i.e. detecting a real QTL, confirming its effect, and obtaining its near-isogenic line-like line (NIL-LL). We demonstrate the feasibility of RapMap by cloning eight rice grain-size genes using 15 F_2_GPs in three years. These genes explain a total of 75% of grain shape variation. Allele frequency analysis of these genes using a large germplasm collection reveals directional selection of the slender and long grains in *indica* rice domestication. In addition, major grain-size genes have been strongly selected during rice domestication. We think application of RapMap in crops will accelerate gene discovery and genomic breeding.

## Abstract (web summary)

Cloning quantitative trait loci (QTL) in crops is often slow and laborious. Here, the authors describe RapMap, a method to rapidly clone multiple QTL based on F_2_ gradient populations coupled with a co-segregation standard, and show how it can be used to identify genes controlling grain size in rice.

## Introduction

Natural variation of food plants and farm animals provides a substantial genetic basis for their domestication and subsequent improvement^1,2^. Agronomically and economically important traits with natural variation in a species are usually controlled by multiple QTL^3–5^. A full understanding of natural variation in a trait requires the identification of the causal genes, which is a long-standing challenge to go from a QTL to its corresponding gene^6–8^. Identifying the functional variations of QTL genes (QTGs) that contribute to the phenotype difference is an important goal for illustrating how basic and applied biology research can be synergistic^3,5,7,8^. During the last two decades, bi-parental populations, including F_2_, recombinant inbred line (RIL), doubled haploid (DH) and backcross (BC) populations, are usually employed for primary QTL mapping, and the map-based cloning approach employing enough recombinants has been proved as the most successful and reliable strategy to isolate the causal QTGs in crops due to the tight linkage of its functional variation with many other variations in a QTL region^8–12^. However, the identification and dissection of a QTL into a single Mendelian factor using the traditional approaches often require the complex construction of advanced generation populations and near-isogenic lines (NILs) by repeated backcrossing, which are rate-limiting steps of cloning a QTL and remain time-consuming and laborious^3,5,9^. Moreover, a very limited number of QTGs identified so far by using some bi-parental populations can only explain partial phenotypic variation in a population^8,10–13^.

Multi-parental populations overcome the limitations of traditional mapping using bi-parental populations and offer an ideal potential to accurately define the genetic basis of complex traits^14^. Genome-wide association studies (GWAS) can be an alternative approach to evaluate the genetic basis of complex traits by looking for associations between SNPs/InDels and phenotypic variation across a large panel of naturally occurring accessions^15,16^. GWAS benefits from high genetic diversity and a historical accumulation of recombination events. However, GWAS requires further analyses with many additional experiments to discover the causal genes, because of the massive population structure, the lower power for rare alleles, and the lower rate of local linkage disequilibrium decay^15,16^. The multiparent advanced generation intercross (MAGIC)^17,18^ and nested association mapping (NAM)^19^ populations offer other alternative approaches to GWAS using natural un-controlled populations and linkage mapping using bi-parental populations. MAGIC and NAM have major advantages in low population structure, balanced parental contributions, abundant genetic diversity, increased recombination, and mapping resolution. However, the development of a MAGIC or NAM population requires a complex design for parent selection, much intercrossing and inbreeding, which are time- and energy-consuming processes^14,17–19^. Currently, MAGIC and NAM populations mainly offer preliminary mapping with high resolution for some loci, and the causal genes of a trait have rarely been identified effectively^14^. Therefore, conventional strategies of QTL mapping and cloning are not only very complex, time-consuming, and costly but also fail to provide a comprehensive understanding of natural variation in a natural population.

Grain size or shape in rice, determined by the three geometrical dimensions (grain length, width, and thickness) with diverse variation, is an important target trait of stable yield, grain appearance, and processing quality, domestication, and breeding. About fifteen QTGs controlling the trait have been identified in the past fifteen years^20–22^. So far, G protein signaling, biosynthesis and signaling of BR, IAA, and CK, peptide signaling, MAPK signaling, the ubiquitin-proteasome degradation pathway, epigenetic pathways, and transcriptional regulation have been identified to be involved in the regulation of grain size in rice^21^. However, due to the very limited number of QTGs identified, the subtle regulatory relationship among QTGs in grain-size variation remains elusive, which may not be addressed based on analysis of artificial mutants and becomes an important research field in agricultural science^7,8^. Therefore, more grain-size QTGs need to be identified to reveal the regulatory mechanism of QTG interactions in rice^4,7,8,20,21^.

Enlargement of specific organs is the most widely emphasized feature among all changes arising from domestication in plants^22–27^. Grain size is an important and typical domestication trait, which distinguished the crops from their wild progenitors. However, how humans domesticated the grain size of rice remains unclear. With the great superiority of deeper seed burial in soil and the feature of easy harvesting under agriculture cultivation, larger grains can germinate more effectively as reproductive organs and provide more nutrients for progenies to produce more vigorous seedlings with greater fitness^26^. As edible organs of many cereal crops, larger grains selected by humans can increase grain yield and provide more food. Archaeological studies of wheat and barley have demonstrated that enlargement of seed size might have arisen during the early history of cultivation but before the completion of crop domestication, and the increase in grain size may be one of the first traits experiencing selection pressure from human cultivation^26^. Domestication signatures include altered allele frequency and a reduction in nucleotide diversity in the domestication loci. Thus, the deep elucidation of the molecular basis for natural variation in domestication traits is essential for bridging molecular analysis of gene function and domestication investigation^3–5,27^. However, the global molecular basis underlying natural variation of grain size in rice is still unclear, which hinders the extensive investigations of domestication at a population level^23–27^.

To address these problems, we introduce a method called Rapid Mapping (RapMap) for easily, rapidly and powerfully cloning QTL associated with traits of interest. It is characterized by the integration of a three-in-one framework under a co-segregation standard. RapMap is also featured by the construction of a series of F_2_ gradient populations derived from multiple bi-parents with gradient phenotypes in diverse germplasms. We apply RapMap to simultaneously discover eight genes controlling the natural variation of grain length and grain width in rice within three years. Besides, in-depth analysis of the eight genes using large and geographically diverse germplasms reveals the domestication signatures of grain-size variation at the population level.

## Results

### Principles of RapMap

**Fig. 1:**
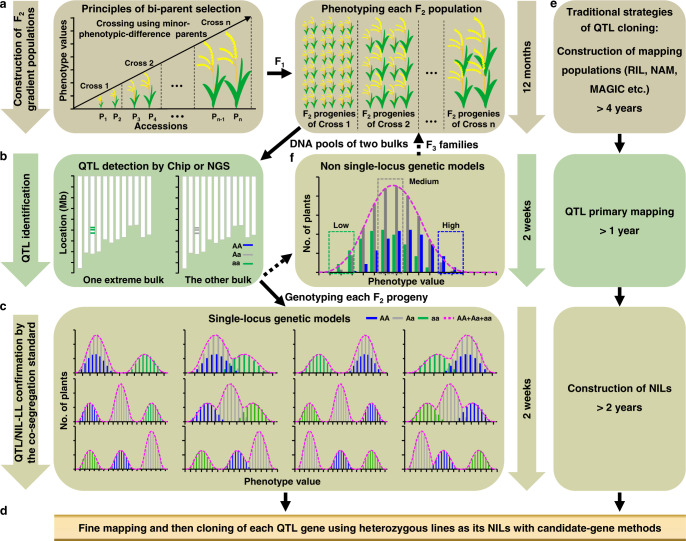
A simplified scheme and principle of RapMap. **a** Construction of F_2_ gradient populations using minor-phenotypic-difference parents from diverse germplasms based on the standard described in the text. Each F_2_GP is generated from self-pollinated F_1_ plants and phenotypes of F_2_ progenies are measured. **b** Identification of QTL. Two DNA pools of high-value and low-value bulks are subjected to microarray chip or next-generation sequencing analysis. **c** The co-segregation standard that the target phenotypes of two homozygous genotypes of a QTL in progenies of any segregating population can be distinguished, is the general principle of the 12 single-locus genetic models as well as the quality-control standard for simultaneous verification of a real QTL and its NIL-LLs. All the individuals from each F_2_GP are genotyped by the markers flanking each candidate QTL region and the co-segregation standard is employed to confirm each QTL and its NIL-LLs simultaneously. **d** Fine mapping and cloning of each QTG by screening enough recombinants using the heterozygous plants of NIL-LLs, coupled with various candidate-gene methods. **e** Traditional processes of QTL cloning that take about eight years and huge efforts. **f** The genetic population that fails to meet the co-segregation standard could be replaced by a phenotype-well-segregating F_3_ or F_4_ family derived from a randomly selected low-, medium- or high-value F_2_ or F_3_ line.

Here, we introduce a method called RapMap, which is efficient and effective for simultaneously discovery of multiple QTL controlling natural variation. As illustrated in Fig. 1, the principle and method of RapMap are explained by taking rice as an example. To clone a QTL, obtaining a mapping population with Mendel’s single gene segregation is the first step. From all the cases of QTL cloning in rice^12^, it can be seen that not more than four QTGs for a specific trait could be isolated in a traditional genetic population constructed by large-phenotypic-difference bi-parents. Therefore, our hypothesis in RapMap is that if only a few QTGs can be identified using large-phenotypic-difference parents, there will be much fewer QTGs (usually only a major one) controlling the relative phenotype of a trait between minor-phenotypic-difference parents. To define the extent of the minor phenotypic difference between parents, we proposed the phenotypic difference index (PDI), the ratio of the difference of phenotypic values between the selected bi-parents to the phenotypic value of the low-value parent. To obtain a mapping population with single-locus control, we first collected a rice mini-core collection with high genetic diversity across the world, phenotyped them for a target trait, divided the accessions into several gradient groups with similar trait values in each group (with PDI <5%) and the minor phenotypic difference between adjacent groups (with PDI from 8% to 25% or a proposed threshold of PDI below 20%), and then selected minor-phenotypic-difference parents (from P_1_ to P_n_) from each gradient group to make a series of gradient crosses (left panel of Fig. 1a), such as P_1_ with P_2_, P_2_ with P_3_, P_3_ with P_4_. The subsequent F_2_ populations derived from these gradient crosses using minor-phenotypic-difference parents are defined as F_2_ gradient populations (F_2_GPs, right panel of Fig. 1a). The construction of a series of F_2_GPs is aimed to minimize the number of segregating loci responsible for the phenotypic variation of a genetic population and maximize the possibility of discovering more QTGs using all populations as much as possible. In addition to the PDI standard of bi-parent selection for constructing F_2_GPs, the cloned genes and the kinship of the parents could also be considered in advance if available. For a target trait without cloned QTGs, we propose to adopt accessions with a relatively higher PDI and closer kinship for crossing. For a target trait with known QTGs, we suggest that the bi-parents for crossing should share the identical/similar alleles of the cloned genes, which could increase the probability of identifying new QTL. More importantly, any F_2_GP developed from any parents should be finally checked by the co-segregation standard in RapMap (see the last paragraph of the part), which is the final guarantee to determine a mapping population with single-locus segregation suitable for rapid QTL cloning.

The crossing number of F_2_GPs for the first round of RapMap depends on the trait variation range of the core collection and the PDI chosen for defining minor-phenotypic-difference parents. In our practices, we have realized that different genes or alleles may be found at a similar gradient level, and the same alleles may be found at different gradient levels. So, in the first round of RapMap, we recommend constructing 6–9 F_2_GPs using minor-phenotypic-difference accessions of diverse phenotypic values for crossing. Alternatively, the minimum number of crosses could be predicted by Eq. 1 detailed in Methods. The predicted numbers of crosses for grain size in this study were close to our recommended 6–9. Then, the total phenotypic variation explained (PVE) in a natural population by these genes identified in the first round of RapMap should be evaluated to determine whether the second round of RapMap is required by making additional crosses using the minor-phenotypic-difference lines with the identical or similar alleles of cloned QTGs.

Because of the minor phenotypic difference of bi-parents determined by PDI, most alleles responsible for the trait in F_2_ are homozygous or function-similar in F_1_, and the number of segregating loci responsible for their phenotypic variation is minimal (a major one in most cases). Unequivocal phenotype segregation caused by one locus can be found in F_2_ progenies and the corresponding QTL can be easily mapped and cloned. Therefore, to detect the QTL region for each F_2_GP, the bulked segregant analysis (BSA) is performed using two phenotype-extreme DNA pools by bulking >30 individuals for each pool from segregating progenies of each F_2_GP, with two phenotype extremes in the top and bottom 15%, respectively^28^. The SNPs or InDels responsible for target phenotypic variation are homozygous in at least one extreme pool (Fig. 1b). Therefore, by subjecting two DNA pools of each population to SNP chip or sequencing, a set of clustered SNPs or InDels between the two pools are very likely to be tightly linked to the causal QTL gene controlling the phenotypic variation (Fig. 1b).

**Fig. 2:**
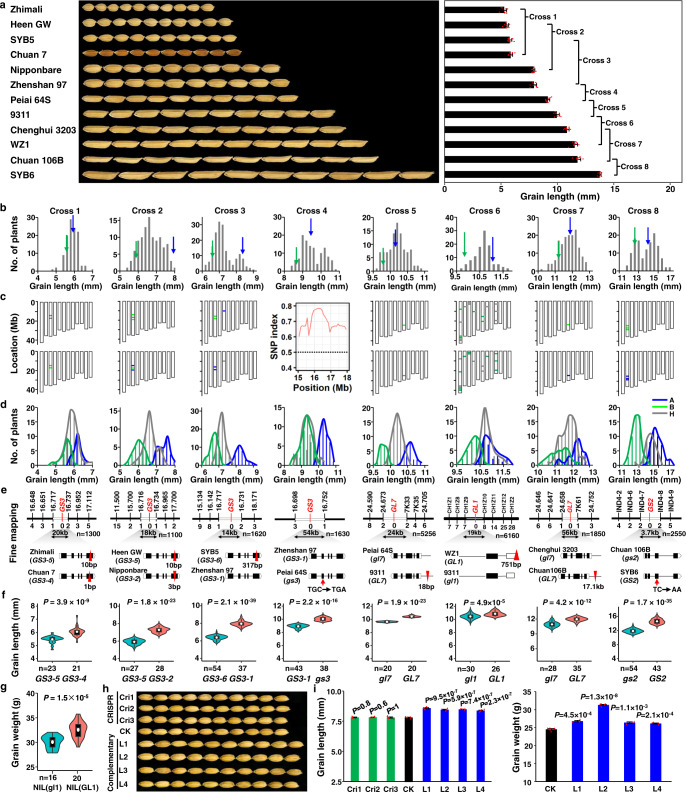
Cloning multiple QTL genes for grain length variation in rice using RapMap. **a** Construction of F_2_GPs using minor-grain-length-difference parents selected from the rice mini-core collection worldwide. The grain length data of each parent is presented as the mean value ± SD (right panel, *n* = 10). Each data point is plotted on the bar (red dots). **b** Distribution of grain length of each F_2_GP. Green and blue arrows indicate low-value and high-value parents, respectively. Top and bottom 15% of the plants were pooled as low-value and high-value bulks, respectively. **c** Identification of grain-length QTL by RICE6K or next-generation sequencing analyses. The QTL for Cross 1–3, and 5–8 were identified by RICE6K, while that of Cross 4 was identified by next-generation sequencing analysis. **d** Confirmation of the candidate grain-length QTL by the co-segregation standard. A (blue), B (green), and H (grey) represent high-value homozygous, low-value homozygous, and heterozygous genotypes of each QTL for each F_2_GP, respectively. **e** Fine mapping and cloning of each grain-length QTL gene using recombinants from corresponding heterozygous NIL-LLs. Black and white bars indicate the chromosome and the QTL interval, respectively. The markers are placed above the chromosomes. The number of recombinants supporting each marker is listed under the chromosomes corresponding to the marker. The gene structures of two parents are presented at the bottom, with boxes and lines representing exons and introns, respectively. Triangles and arrows in red indicate functional insertions and SNPs, respectively. **f** Confirmation of the genes by comparing grain length between two homozygous NIL-LLs identified by functional markers of cloned genes. **g** Grain weight of NIL(WZ1) and NIL(9311) for the grain-length gene *GL1*. Data are presented as violin plots embedded with box plots which displayed the density distribution (violin), the minima (bottom), maxima (top), center (line in the middle of boxes), mean (white circle), bounds of the box and whiskers (vertical line). **h** Phenotypes of CRISPR lines (Cri1 to Cri3), a negative line (CK), and four positive complementary lines (L1 to L4) for the *GL1* gene. **i** Statistics of grain length or weight of CRISPR lines, negative lines, and complementary lines (*n* = 15) for *GL1*. All data are shown as mean value ± SEM. The two-tailed student’s *t*-test was used to generate *P* values. *n* represents the number of biologically independent experiments or samples. Source data underlying Figs. 2a, b, d, f, and g are provided as a Source Data file.

**Fig. 3:**
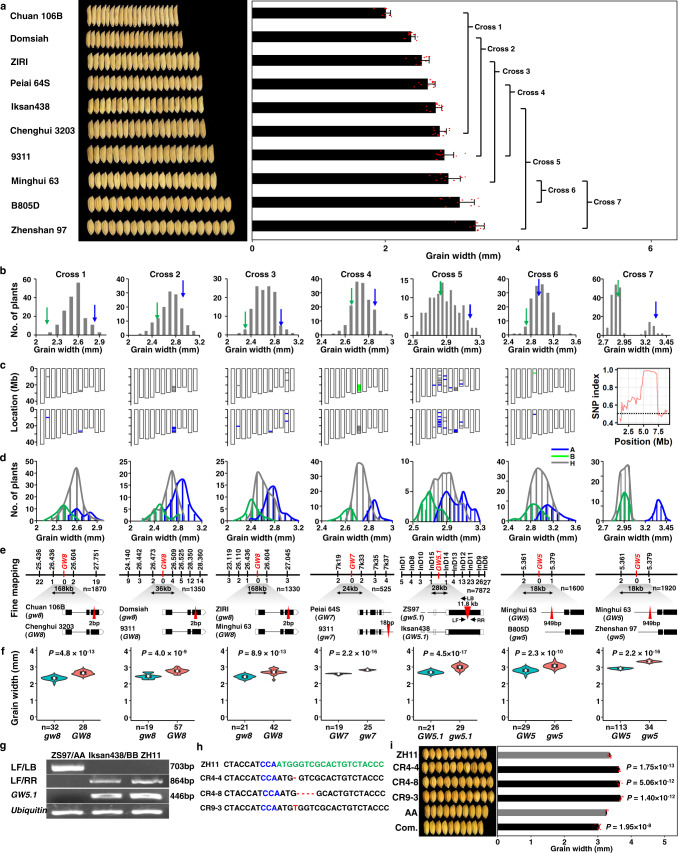
Identification of multiple QTL genes for grain-width variation in rice using RapMap. **a** Construction of F_2_GPs with minor-grain-width-difference parents selected from the rice mini-core collection. The grain width data of each parent is presented as the mean value ± SD (right panel, *n* = 10). Each data point is plotted on the bar (red dots). **b** Distribution of grain width of each F_2_GP. Green and blue arrows indicate low-value and high-value parents, respectively. Top and bottom 15% of the plants were pooled as low-value and high-value bulks, respectively. **c** Identification of grain-width QTL by RICE6K or next-generation sequencing analyses. The QTL of Cross 1–6 were identified by RICE6K, while that of Cross 7 was identified by next-generation sequencing analyses. **d** Confirmation of the candidate grain-width QTL by the co-segregation standard. A (blue), B (green), and H (grey) represent high-value homozygous genotypes, low-value homozygous genotypes, and heterozygous genotypes of each QTL for each F_2_GP, respectively. **e** Fine mapping and cloning of each grain-width QTG using recombinants from corresponding heterozygous NIL-LLs, coupled with various candidate-gene methods. The black and white bars indicate the chromosomes and the QTL intervals, respectively. The markers are placed above the chromosomes. The number of recombinants supporting each marker is listed under the chromosome corresponding to the marker. The gene structures of two parents are drawn at the bottom, with boxes and lines representing exons and introns, respectively. Triangles in red indicate functional insertions. **f** Confirmation of the genes by comparing grain width between two NIL-LLs identified by functional markers of cloned genes. Data are presented as violin plots embedded with box plots which displayed the density distribution (violin), the minima (bottom), maxima (top), center (line in the middle of boxes), mean (white circle), bounds of the box and whiskers (vertical line). **g** Validation of the 11.8 kb LTR-retrotransposon insertion at DNA and mRNA levels. Each experiment was repeated independently three times and all showed similar results. **h** The gRNA target site (marked in green) and the resulting frameshift mutations (marked in red) in the ORF1 gene of the three independent CRISPR plants (CR4-4, CR4-8, and CR9-3). PAM sequences are marked in blue. **i** Functional verification of *GW5.1* by CRISPR and complementation transformation. Com. is the positive complementary line; ZH11 and AA are the control of the CRISPR and complementation transformation, respectively. All data are represented by mean value ± SEM (*n* = 10). The two-tailed student’s *t*-test was used to generate the *P* values. *n* represents the number of biologically independent experiments or samples. Source data underlying Fig. 3a, b, d, f, g, and i are provided as a Source Data file.

To find a quality-control standard for confirming a mapping population with a single-locus segregation pattern, we have updated the concept of Mendel’s single gene segregation into 12 single-locus genetic models in RapMap according to three different genetic effects (complete dominant, semi-dominant, and over-dominant) (Fig. 1c): (1) an allele could be low-value or high-value complete dominant for the phenotypic distribution with a discrete (no environmental or other effects) or partially overlapping (due to environmental or other effects) boundary and a ratio of 3:1 or 1:3 (upper panel of Fig. 1c); (2) an allele could be semi-dominant or additive for the phenotypic distribution with two discrete or partially overlapping boundaries and a ratio of 1:2:1 (Middle panel of Fig. 1c); (3) an allele could be over-dominant for the phenotypic distribution with two discrete or partially overlapping boundaries and a ratio of 1:1:2 or 2:1:1 (lower panel of Fig. 1c). Through insightful induction of the 12 single-locus genetic models, we discover they share the common and general nature (called the co-segregation standard hereafter) that the two homozygous genotypes (AA and aa) of a target QTL coincidently co-segregate with their corresponding phenotypes, regardless of the heterozygous genotypes (Fig. 1c). In the traditional judgment in literature, the control of a single-locus and multiple loci is determined by a bimodal (or 3:1) and normal distribution of progeny phenotypes, respectively, which should be updated to the co-segregation standard. The co-segregation standard is also a quality-control standard for the simultaneous verification of a candidate QTL and its NIL-LLs in RapMap. If two homozygous genotypes of a candidate QTL region determined by both flanking molecular markers in progenies from any randomly segregating population can co-segregate with their corresponding phenotypes, the locus is confirmed as a real QTL for this trait. The heterozygous lines for the QTL in each F_2_GP are the corresponding NIL-LLs suitable for map-based cloning of the QTL coupled with various candidate-gene methods (Fig. 1c and d). Therefore, mapping a QTL, verifying its effect, and obtaining its NIL-LLs, three decisive rate-limiting steps for the cloning of a QTL in traditional methods, are integrated into a three-in-one step in RapMap (Fig. 1a–d), which is ensured by the co-segregation standard (Fig. 1a–d), greatly improving the efficiency and accuracy of QTL identification compared with traditional methods (Fig. 1e). The genetic population that does not meet the co-segregation standard can be replaced by a different F_2_GP constructed using bi-parents at a similar gradient level, or by a phenotype-well-segregating F_3_ or F_4_ family derived from the selfing of a randomly selected low-, medium- or high-value F_2_ or F_3_ line, to make minor-effect loci homozygous and one major-effect locus heterozygous through checking the phenotype segregation (Fig. 1f). To sum up, the co-segregation standard, which serves as the necessary and sufficient condition of single-locus genetic models, is the cornerstone of RapMap, while the construction of multiple F_2_GPs to most probably meet the quality-control standard is its technical strategy. Thus, the co-segregation standard coupled with multiple F_2_GPs ensures that RapMap can be applied to any plants and animals that are easy to crossbreed and reproduce.

### Identification of four QTL genes for rice grain length by RapMap

As a proof-of-principle experiment, we applied RapMap to map and isolate QTGs for natural variation of grain length and width in rice. We have collected, maintained, and sequenced a rice mini-core collection of more than 541 accessions, including both landraces and improved varieties from 59 countries, representing the high genetic diversity in this species^29,30^ (Supplementary Data 1). For grain length, we selected 12 gradient parents, whose grain length ranged from 5.58–13.76 mm, and developed eight F_2_GPs using these parents with the average PDI of 16.6% (the range from 10.3%–30.4%) within 12 months (Fig. 2a). After measurement of grain length of about 200 F_2_ progenies of each population, we constructed a high-value bulk (about the top 15%) and a low-value bulk (about the bottom 15%) for each population (Fig. 2b). Following the rapid DNA extraction, two bulked DNA pools for each of the seven populations (Cross 1–3 and 5–8) were subjected to the commercial rice whole-genome SNP arrays (RICE6K)^31^. SNPs detected by the SNP arrays between two extreme pools were tightly linked to the causal QTG for grain-length variation (Fig. 2c). Alternatively, following the principle of SNP index estimation in QTL-seq^32^, we performed whole-genome sequencing of two DNA bulks from Cross 4 progenies and identified a cluster of SNPs with an SNP index close to 1 for the target locus (Fig. 2c), but the index was nearly 0.5 for the other loci (Supplementary Fig. 1). Therefore, the SNP cluster was most likely near the causal QTG. To confirm the candidate QTL, verify its effect and select its corresponding NIL-LLs suitable for map-based cloning, we first designed 2–4 InDel markers in a 2–4 Mb region that covers each candidate QTL region (Fig. 2c) with the database of rice genomic variations^33^, and then genotyped all the individuals of each F_2_GP using the InDel markers linked with the locus. Finally, each F_2_GP was checked by the co-segregation standard and the two boundaries of each QTL were confirmed based on the recombinant events (Fig. 2d). If the co-segregation standard was satisfied (Fig. 2d), the SNP region was verified as a real QTL for grain-length variation, and the heterozygous lines of the QTL region could be the corresponding NIL-LLs. In fact, the Cross 5 population did not meet the co-segregation standard of single-locus genetic models in F_2_ and was replaced by a grain-length-well-segregating F_4_ family derived from a selected medium-value F_3_ line (Figs. 1f, 2). Finally, we verified each QTL for each F_2_GP and confirmed the minimum QTL regions on chromosomes 3, 3, 3, 3, 7, 1, 7, and 2 for Cross 1 to Cross 8, respectively (Fig. 2e). The PVE of each QTL in the corresponding F_2_GP was 44%, 85%, 90%, 79%, 94%, 50%, 58% and 83%, respectively (Supplementary Table 1).

To fine map each of the above grain-length QTL identified by RapMap, sufficient recombinants in progenies of corresponding heterozygous NIL-LLs were screened using the KASP high-throughput genotyping technique^34^ based on the two flanking markers and the rapid DNA extraction technique in the greenhouse. Based on both high-density marker genotypes and grain-length phenotypes of these recombinants, we located the grain-length QTL to the regions of 20, 18, 14, 54, 24, 19, 56, and 3.7 kb for Cross 1 to Cross 8, respectively, in which there is only one to three predicted open reading frames (ORFs) (Fig. 2e). To determine whether the known grain-length gene in each fine-mapping region is the causal gene, comparative sequencing analyses of their alleles between the two parents were conducted. As a result, the known functional variations of all three grain-length genes (*GS3*, *GL7,* and *GS2*) reported in references^35–40^ were indeed discovered in Cross 1 to Cross 8, except for Cross 6 (Fig. 2e). Thus, there was no need to reassess a known gene underlying the QTL with the same functional variation using a transgenic approach. Self-pollination of the heterozygous NIL-LLs for each QTL produced two homozygous NIL-LLs, and the two homozygous genotypes determined by the reported functional marker of each cloned gene could distinguish long-grain and short-grain progenies very well (Fig. 2f), further confirming the three causal genes for grain-length variation in F_2_GPs.

Two previously unknown alleles of the major grain-length gene *GS3* (*GS3-5* from Cross 1 and Cross 2, and *GS3-6* from Cross 3) were identified (Fig. 2 and Supplementary Fig. 2). The *GS3-5* allele exhibited a stronger function (shorter grain) than the *GS3-4* allele from Chuan 7 that has been reported to have the strongest function so far (Cross 1 of Fig. 2f and Supplementary Fig. 2a)^37^. The *GS3-6* allele has a stronger function (shorter grain) than that of the *GS3-1* allele (Cross 3 of Fig. 2f and Supplementary Fig. 2a). Compared with the four known alleles of *GS3*^37^, *GS3-5* and *GS3-6*, with a 10 bp and 317 bp deletion in the last exon, respectively, have different functional variations with reported ones, resulting in two much shorter frameshifts of the C terminal than the *GS3-4* allele (Fig. 2, Supplementary Fig. 2b, c).

A previously unreported grain-length gene *GL1* was discovered in the RapMap programs using the Cross 6 population (Fig. 2). The only ORF encoding a cytochrome P450 protein (LOC_Os01g63930) in the 19 kb fine-mapping region (Fig. 2e) was determined as the reliable candidate gene of *GL1*. We sequenced the 2.3 kb promoter region, the coding region, and the 3′UTR of *GL1* in WZ1, 9311, and Zhonghua 11 (ZH11), and found many variations among them (Supplementary Fig. 3a). Compared with ZH11, WZ1 has a 2-bp insertion and 9311 has a 751-bp deletion at the 3′UTR. Three major haplotypes of *GL1* were identified in the rice mini-core collection based on variations in their promoters and 3’UTR regions. The relative expression level of *GL1* in the WZ1 haplotype (with longer grains) was higher than that in both 9311 and ZH11 haplotypes (with shorter grains) (Supplementary Fig. 3b). No difference in the expression level was observed between the 9311 and ZH11 haplotypes (Supplementary Fig. 3b). These results were further confirmed by transient expression assays using their promoters and 3′UTR regions. 9311 and ZH11 fragments showed comparable activity, which was much lower than that of the WZ1 fragments (*P* ≤0.01) (Supplementary Fig. 3c). Thus, the alleles of *GL1* in both ZH11 and 9311 have a similar lower expression level with a short-grain function, while it is the opposite case in WZ1, with the dominance feature of the *GL1* allele as revealed by the F_2_ population analysis (Fig. 2d). Self-pollination of the BC_4_F_1_ plants heterozygous at this locus generated two NILs in the 9311 background, NIL(WZ1) and NIL(9311). Compared with those of NIL(9311), the grains of NIL(WZ1) exhibited 7.4% longer in length and 8.3% heavier in weight (Fig. 2g and Supplementary Fig. 3d). To further confirm the function of the candidate gene for *GL1*, we generated the constructs for gene editing by CRISPR and complementation transformation. Considering the difficulty in regenerating plants from the *indica* calli of both WZ1 and 9311, the dominance of the WZ1 allele and the similarity of the two alleles from both 9311 and ZH11 (Fig. 2 and Supplementary Fig. 3b–c), we performed genetic transformation using the *japonica* variety ZH11 as acceptors. First, the *GL1* allele in the ZH11 background was knocked out by CRISPR (Supplementary Fig. 3e), and 20 positive lines out of 26 transgenic plants were generated. No significant differences in grain length were found between negative and positive plants when we knocked out the *GL1* allele in ZH11 (Fig. 2h, i and Supplementary Fig. 3e, f), further suggesting that the *GL1* allele in ZH11 or 9311 was of no or weak function. Thus, we transformed the complementation vector containing the coding region of the dominant *GL1* allele from WZ1 driven by its 2.3 kb native promoter into ZH11. Thirty out of the 36 complementation plants were positive lines, and most of them exhibited increases in grain length and grain weight (Fig. 2h, i and Supplementary Fig. 3g). Co-segregation tests of the T_1_ progenies from four T_0_ plants showed that the grain length co-segregated with the transgene (Supplementary Fig. 3h). Taken together, these results suggest that the only ORF in *GL1* serves as the regulator of grain size.

### Cloning of four QTL genes for rice grain width by RapMap

We also applied RapMap to the cloning of QTGs for rice grain width (Fig. 3). First, we selected 10 gradient parents, whose grain width varied from 1.99–3.43 mm and PDI ranges from 5.8%–30.7% (the average PDI is 18.3%), and constructed seven F_2_GPs using these parents (Fig. 3a). It should be noted here that if the difference in grain width between the two parents was not very big, the cross would be compatible and suitable for RapMap (Fig. 3a). Similar to the investigation of grain length, after RICE6K array or genome sequencing analyses of bulked DNA pools of grain-width populations (Fig. 3b, c and Supplementary Fig. 4), seven real QTL from seven F_2_GPs were determined by the co-segregation standard, and were located on chromosomes 8, 8, 8, 7, 5, 5, and 5 for Cross 1 to Cross 7, respectively (Fig. 3d). The PVE of each QTL in each F_2_GP population was 61%, 51%, 58%, 90%, 60%, 53%, and 93%, respectively (Supplementary Table 2). To clone each grain-width QTG, we screened sufficient recombinants for each real QTL. Finally, using the marker genotypes and the grain-width phenotypes of these recombinants, we located the grain-width QTL to regions of 168, 36, 168, 24, 28, 18, and 18 kb for Cross 1 to Cross 7, respectively, which contained few ORFs (Fig. 3e) for each region after the candidate-gene analysis of the two bigger fine-mapping regions. To determine whether the known grain-width genes *GW8*, *GW7,* and *GW5* within these regions are the causal genes, their known functional variations^41–45^ were finally validated by comparative sequencing analyses of their alleles (Fig. 3e). Their corresponding NIL-LLs further confirmed the three causal genes (Fig. 3f).

A previously unknown grain-width gene *GW5.1* was identified in the RapMap process using the Cross 5 population (Fig. 3). In the 28 kb fine-mapping region, only two ORFs were annotated by the RAP-DB website. We then identified their full-length cDNAs corresponding to ORF1 and ORF2 (NCBI accessions AK121364 and AK065965, respectively), and searched the public expression database of Zhenshan 97 and Nipponbare (http://crep.ncpgr.cn/; http://ricexpro.dna.affrc.go.jp/). As a result, very low expression of ORF2 was detected in young panicles. Moreover, the second exon of the ORF1 allele from Zhenshan 97 or the AA genotype was disrupted by an 11.8 kb LTR-retrotransposon insertion, as indicated by comparative sequencing of the two alleles from the two parents (Fig. 3, Supplementary Fig. 5a), which results in the function loss of the ORF1 allele (Fig. 3g). Compared with NIL(*GW5.1*), the grains of NIL(*gw5.1*) were 14.9% wider in width and 12.9% heavier in weight (Supplementary Fig. 5b). Therefore, ORF1 encoding a receptor-like kinase (LOC_Os05g25350) was determined as the reliable candidate gene of *GW5.1*. To validate the candidate gene, CRISPR and complementation transformation strategies were carried out. All the three independent frameshift mutants produced wider grains than ZH11, a *japonica* variety with the same functional allele as Iksan438 (Fig. 3h, i and Supplementary Fig. 5c, d). Consistent with mutant results, the positive plant carrying the complementation vector produced narrower grains than AA (Fig. 3i), suggesting that ORF1 was a negative regulator of grain width in rice. Co-segregation tests of progenies from the complementation line showed that the grain width co-segregated with the transgene (Supplementary Fig. 5e). Taken together, these data indicate that ORF1 is the right candidate for *GW5.1*.

### Genetic contributions of the eight genes to grain size/shape in a rice mini-core collection

**Fig. 4:**
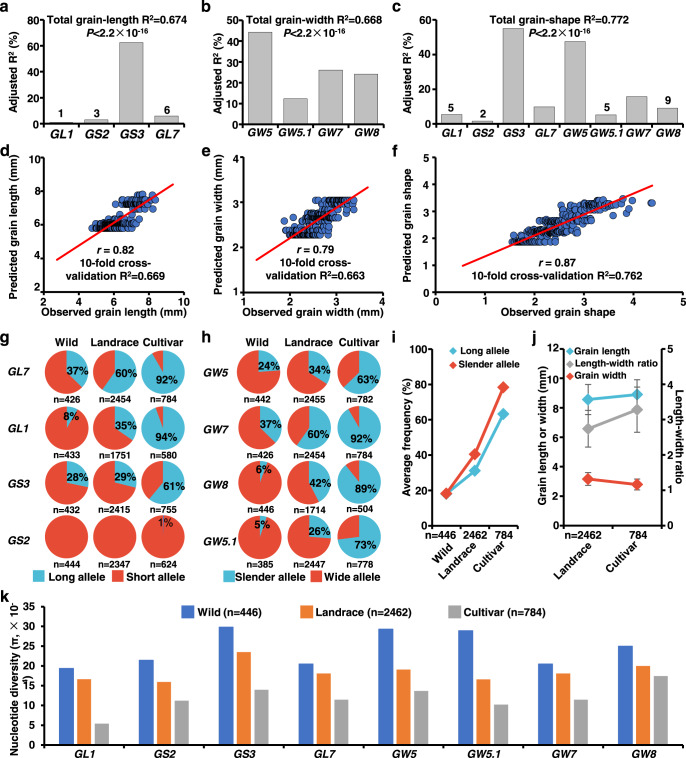
Genetic contribution and directional selection of eight grain-size genes during rice domestication and improvement. **a–c** Contribution and relative importance of the identified genes for grain length (**a**), grain width (**b**) and grain shape (**c**) of brown rice in the rice mini-core collection with 541 accessions. The phenotype variance explanation (R^2^), which was determined by the multiple linear regression of the Eq. (2) with the genotypes of the functional markers of eight genes as the predictor variables, was used to assess the genetic contribution of these genes. R^2^ above the bars represents the total contribution of the genes for grain length (**a**), grain width (**b**) and length-width ratio (**c**). Each bar indicates the contribution of one individual gene to the trait. The exact *P* values of four grain-length genes are 5.6 × 10^−2^, 4.5 × 10^−2^, 2.2 × 10^−16^, and 3.8 × 10^−7^, respectively (**a**). The exact *P* values of four grain-width genes are <2.2 × 10^−16^, 9.9 × 10^−12^, <2.2 × 10^−16^ and <2.2 × 10^−16^, respectively (**b**). The exact *P* values of eight grain-size genes are 4.6 × 10^−6^, 1.1 × 10^−2^, <2.2 × 10^−16^, 4.2 × 10^−11^, <2.2 × 10^−16^, 1.8 × 10^−5^, <2.2 × 10^−16^, and 5.6 × 10^−9^, respectively (**c**). **d**–**f** The power of the prediction model for grain length (**d**), grain width (**e**) and grain shape (**f**). 10-fold cross-validation was used to estimate the model prediction performance. **g**, **h** Frequencies and directional selection of alleles of grain-length (**g**) and grain-width (**h**) genes within the three germplasm groups with 446 wild, 2462 landrace and 784 cultivar accessions of rice. **i** Average allele frequency of long-grain and slender-grain alleles from wild to landrace and from landrace to cultivar. **j** Average grain length, grain width and the length-width ratio of the landraces and cultivars. Data are presented as mean ± SD. *n* shows the biologically independent samples. **k** Nucleotide diversity of eight grain-size gene loci (2 Mb) in wild, landrace and cultivar accessions of rice. Source data underlying Fig. 4a–f and 4i–k are provided as a Source data file.

To assess the power of RapMap, we investigated the effect size and predictive ability of the eight genes cloned using a single round procedure of RapMap. Using the genotypes of the functional variations of the eight genes and the phenotypes of 541 accessions, we performed a multiple linear regression analysis to assess the genetic contributions of the eight genes to grain size and grain shape (length-width ratio) in the rice mini-core collection (Fig. 4a–f and Supplementary Data 1). The four grain-length genes (*GL1*, *GS2*, *GS3,* and *GL7*) and four grain-width genes (*GW5*, *GW5.1*, *GW7,* and *GW8*) explained 67.4 and 66.8% of the phenotypic variation in the rice mini-core collection, respectively (Fig. 4a, b). Among these genes, *GS3* and *GW5* accounted for 62.5% of the grain-length variation and 44.3% of the grain-width variation, respectively, while other genes showed diverse effects on grain length or width (Fig. 4a, b). Because grain shape is an important quality trait of rice, we also investigated the contributions of these eight genes to grain shape (Fig. 4c). The eight genes could explain a total of 77.2% variation of grain shape, among which *GW5* and *GS3* explained 47.5 and 55.0% of the phenotypic variation, respectively (Fig. 4c). We then investigated the prediction performance of the grain-size genes based on their functional variations (Fig. 4d–f). The correlation coefficient between the observed phenotypes and the predicted values was 0.82 for grain length (Fig. 4d), 0.79 for grain width (Fig. 4e), and 0.87 for grain shape (Fig. 4f), showing very high predictive power. In short, through one single round of RapMap with 15 F_2_GPs, more than two-thirds of the genetic variation could be revealed for rice grain size and shape, demonstrating the huge potential of RapMap in the gene discovery and comprehensive elucidation of the molecular basis for a complex trait in rice.

### Directional selection of grain-size genes during rice domestication and improvement

To reveal the domestication signatures of grain-size variation at a population level, here, we first investigated the allele frequencies of eight grain-size genes identified by RapMap in a large and geographically diverse germplasm collection^46^, including 446 wild, 2462 landrace and 784 cultivar accessions of rice (Supplementary Data 3 and 4). For each of the eight genes, we discovered the stepwise accumulation of all the long-grain alleles (Fig. 4g) and all the slender-grain alleles (Fig. 4h) from wild to landrace and from landrace to cultivar during rice domestication and improvement (Fig. 4i), indicating the directional selection and enrichment of preexisting genetic variants rather than mutations after domestication for all genes except for *GS2*. The strength of directional selection of these long- or slender-grain alleles in *indica* subspecies is much stronger than that in *japonica* subspecies, especially for the major-effect genes *GS3*, *GW5,* and *GL7*/*GW7* (Supplementary Fig. 6a–d). Averagely, the grains of rice cultivars are much longer and slenderer than those of rice landraces, and it is the same case for grain shape (Fig. 4j). These results are consistent with the nucleotide diversity reduction detected for each grain-size locus from wild to landrace and from landrace to cultivar (Fig. 4k). The results demonstrated the enrichment of preferred alleles of all grain-size genes coupled with a reduction of their nucleotide diversity during rice domestication and improvement.

To understand the phenotypic effect of different allele combinations (haplotypes) of the eight grain-size genes and facilitate genomic breeding, we identified nine haplotypes for grain length (Supplementary Fig. 7a) and 16 haplotypes for grain width (Supplementary Fig. 7b) in rice landraces and cultivars based on their functional variations. The grain length of the three haplotypes with the largest frequency in cultivars, including Hap-L7 (78%, 9.50 mm), Hap-L8 (83%, 8.98 mm) and Hap-L9 (100%, 9.05 mm), was higher than the average value (8.56 mm) in landraces (Supplementary Fig. 7a). The grain width of the three haplotypes with the largest frequency in cultivars, including Hap-W14 (35%, 2.80 mm), Hap-W15 (50%, 3.08 mm) and Hap-W16 (50%, 2.78 mm), was lower than the average value (3.16 mm) in landraces (Supplementary Fig. 7b). The length-to-width ratio of all these six haplotypes in cultivars was much higher than the average level in landraces (Supplementary Fig. 7a, b). It should be noted that the haplotypes with the largest frequency in cultivars predominantly comprised *indica* (but not *japonica*) subspecies, which had much slenderer and longer grains than those in landraces (Supplementary Fig. 7c–e). The slender- and long-grain haplotypes and *indica* subspecies are enriched in low-latitude regions with warm temperatures, while it is the opposite for the wide- and short-grain haplotypes and *japonica* subspecies (Supplementary Fig. 8a–c). The landraces broadly distributing worldwide include most *japonica* rice, while cultivars distributing most in low to moderate latitude regions include most *indica* rice (Supplementary Fig. 8d). These observations suggested that rice grains gradually shifted toward slenderer- and longer- grain shape in *indica* subspecies, which might be due to the preferential and nonparallel combinatorial selection and differential geographical distribution of these grain-size alleles in *indica* and *japonica* rice (Supplementary Fig. 6–8). Furthermore, these results provided a basis as well as flexibility for the selection of diverse grain shapes in rice.

### Selection sweeps of grain-size loci and correlations among their selection strength, DNA variations and grain-size effects

**Fig. 5:**
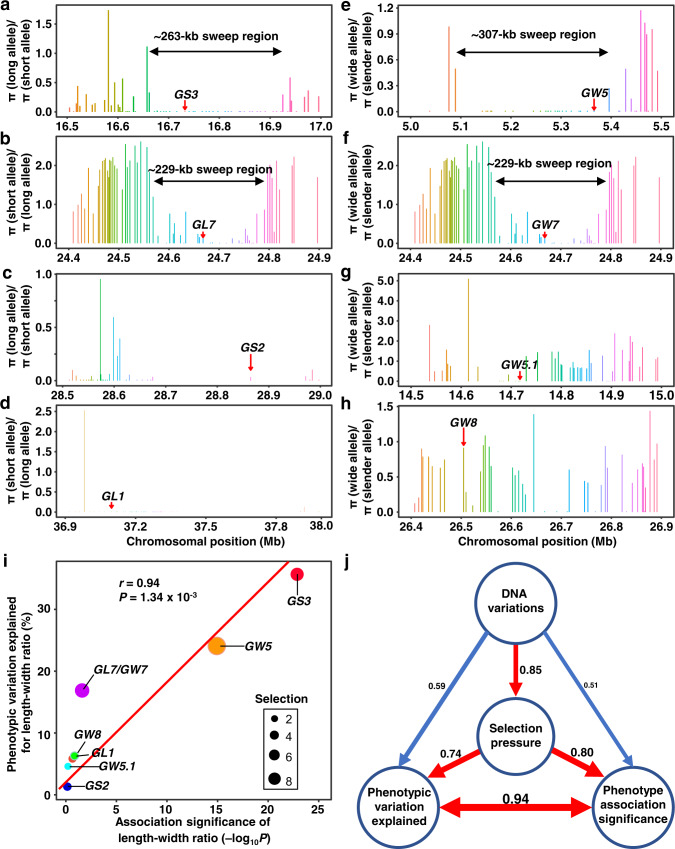
Selection sweeps of the eight grain-size loci in rice cultivars and relationship among their selection strength, DNA variations and grain-size effects. **a–h** Selection sweep scanning across *O. sativa* genome regions of the eight grain-size gene loci in rice cultivars of 784 accessions. Bars indicate the ratios of silent-site nucleotide diversities of each gene in these loci between long- and short-grain allele accessions or wide- and slender-grain allele accessions. The smallest bars are equivalent to a ratio of 0. Red arrows indicate each grain-size gene. **i**, **j** Correlations among DNA variations, selection pressure and phenotype effects of the eight grain-size genes. The PVE for the length-width ratio is the R^2^ used in (Fig. 4c). Association significance of the length-width ratio is the minus log-transformed *P*-value of GWAS for the length-width ratio. The selection pressure is the π ratio of cultivar versus landrace for each gene using VCFtools. DNA variations are the changes in DNA of each gene by comparing the gene sequences of the cultivars and the wild species *O. rufipogon* W1943 using MEGA X (**j**). The arrow width and the number font size are scaled to the size of the correlation coefficients (**j**). Source data underlying Figs. 5i, j are provided as a Source Data file.

To find the selection evidence of the grain-size genes, we scanned each sweep region generated by the linkage disequilibrium of the selection target and its surrounding loci (Fig. 5a–h and Supplementary Fig. 9), which is expected to affect the genetic diversity. By comparing the ratio of the silent-site nucleotide diversity of each gene in a 500 kb genome region of the *GS3* locus between long- and short-grain allele accessions in the cultivar population (Fig. 5a–h), we discovered a distinct valley (spanning ~263 kb) of reduced nucleotide diversity for *GS3*, which has the greatest contribution to grain length, among accessions carrying the long-grain *gs3* allele (Fig. 5a). For *GW5*, the greatest contributor to grain width, there was a ~307 kb valley with reduced nucleotide diversity among the accessions carrying the wide-grain *gw5* allele (Fig. 5e), which has been recognized as a domestication-related gene located at a 4.8 Mb selection sweep by comparing the nucleotide diversity of the wild and cultivated rice^46^. Similarly, there was a typical valley (spanning a ~229 kb region) for *GW7*/*GL7*, the second important gene for grain width and grain length (Fig. 5b, f). However, no evident valley of nucleotide diversity and linkage disequilibrium was discovered for other genes with minor grain-size effects (Fig. 5c–d, g–h and Fig. 4a–c).

To confirm whether this reduction of nucleotide diversity across the target region of *GS3*, *GW5* and *GW7*/*GL7* is an artifact of demographic history, we compared the long-to-short and wide-to-slender π ratios of the target gene loci. The average π ratios of *GS3*, *GW5* and *GW7*/*GL7* within their corresponding 500 kb regions were 0.14, 0.27, and 0.42, which were significantly (*P* < 2.2 × 10^−16^) lower than those at the whole chromosome level (0.74, 0.85, and 1.73), respectively. Thus, it is artificial and directional selection rather than demographic factors that have played an important role in the reduction of nucleotide diversity in these grain-size loci during rice domestication. In addition to cultivar accessions, we also investigated the selection sweeps of these gene loci in the wild rice (Supplementary Fig. 9a–h) and landraces (Supplementary Fig. 9i–p). As a result, for *GS3*, *GW5*, *GW7*, *GS2,* and *GL1*, the genetic diversity gradually decreased along with the domestication process from wild to landrace or cultivar, while *GW5.1* and *GW8* display no obvious decrease (Fig. 5a–h and Supplementary Fig. 9). The selection sweeps of the major genes *GS3*, *GW5,* and *GW7*/*GL7* have been shaped during the landrace period, indicating that their selection signatures have arisen during the early domestication (Fig. 5a, e and Supplementary Fig. 9a, i, e, m). Taken together, these results show the selection strength on the eight genes is likely related to their phenotypic effect size in the large diverse population.

To further understand the relationship among the phenotypic effect size, phenotype association significance, selection strength and nucleotide variations during grain-size domestication and improvement, we analyzed the pairwise correlation of these four aspects for the eight grain-size genes (Fig. 5i, j). The results demonstrated strong positive correlations among these four aspects. Thus, it suggested that important mutations in grain size genes generate abundant phenotypic variation that acts as the target of artificial selection. GWAS using the same rice mini-core collection identified the major *GS3* locus with a large 357.5 kb region and a minor-effect signal at the *GW5* locus for grain length and shape, and the major *GW5* locus with a 132.1 kb region for grain width and shape each coupled with a minor-effect signal (Supplementary Fig. 10). The high correlation between the phenotype association significance with the PVE (Fig. 5i, j, *r* = 0.94) indicated the QTL identified by GWAS always had a great effect on grain size at a natural population level. Alternatively, RapMap can complement shortcomings of GWAS and identified rare alleles or minor-effect genes (such as *GS2* and *GL1*) and moderate-effect genes (*GW8*, *GL7,* and *GW7*) as well (Supplementary Fig. 10). The genes such as *GW5*, *GS3,* and *GW7*/*GL7* located in stronger selection sweep regions (Fig. 5a–h) resulting from their stronger artificial selection pressure, had greater genetic contributions to the whole grain shape variation (Fig. 5i). Simultaneously, these genes usually have more or stronger DNA mutations (Fig. 5j, *r* = 0.85). Although *GS2* has little contribution to the whole phenotype variation in the large natural population due to its rare allele and weak selection (Fig.4g and Fig. 5i), it has a significant effect on grain length in bi-parental linkage populations (Fig. 2 and Supplementary Table 1)^39,40^. However, the favored natural variation of *GS2*, which is already present in cultivars, has also been identified by RapMap. Collectively, we conclude that selection pressure on grain-size genes provides more chances for humans to pick out and easily identify greater-effect genes by association analysis, and the rare or minor-effect genes can be effectively identified by RapMap. Both of the rare and common alleles identified by RapMap provide a great opportunity to investigate the directional selection of grain size and shape during rice domestication and improvement.

## Discussion

We establish a strategy and a quality-control standard for QTL mapping and cloning in RapMap, which can rapidly identify QTL and isolate QTGs responsible for a given trait on a large scale (Fig. 1). RapMap has several notable advantages over traditional schemes of linkage and association analyses (F_2_, RIL, DH, GWAS, NAM, and MAGIC)^7–19^ and those recently reported methods (QTL-seq, QTG-seq, MutMap, and others)^32,47–50^. These advantages include the flexibility of parent selection, the simplicity of constructing multi-parental F_2_GPs, high mapping power and resolution, high genetic diversity to achieve multi-loci mapping with higher reliability and higher PVEs, mapping QTL of minor effect or low allele frequency, fast gene discovery using NIL-LLs, and the three-in-one framework integrated by the co-segregation standard.

Multiple F_2_GPs in RapMap represent a compromise or complementary characteristic between the extreme simplicity of a diallelic system found in an F_2_, RIL, or DH panel, and the great complexity encountered in natural accessions for GWAS and other multi-parental populations (such as MAGIC and NAM)^14^. Most QTL studies have either employed simple bi-parental populations with restricted allelic variations or performed association mapping using naturally occurring haplotypes or other multi-parental populations with much complex construction. Each of these methods has some limitations, therefore, alternative approaches for the genetic dissection of complex traits continue to be sought recently^8,11,14,15,32,47–50^. Here we describe one such alternative, F_2_GPs in RapMap. The F_2_GPs serve as a multi-parental population to greatly improve the efficiency of detecting and cloning multiple major QTL following single-locus segregation, which could not be easily achieved by other methods.

Most genes underlying all the QTL identified by large-phenotypic-difference bi-parents have not been discovered in rice, which is probably due to their minor or artifact effects^8,11–13^. The few QTL genes cloned in rice are usually major ones at bi-parental population level^8,12^. The current findings suggest that the number of major QTL genes controlling the relative phenotype difference of a specific trait between bi-parents is likely very small, and different QTL genes for a trait are usually identified by using different bi-parental populations. Therefore, the rationale of RapMap is based on the hypothesis that there will be much fewer QTGs (usually only a major one) controlling the relative phenotype of a trait between parents with a minor phenotypic difference than those with a large-phenotypic difference. That is why we can construct a series of F_2_GPs by using minor-phenotypic-difference parents from diverse germplasms to most probably obtain mapping populations with single-locus segregation, which is an attempt to reduce the usual genetic complexity behind quantitative traits and easily realize one-QTL-at-a-time mapping and cloning.

RapMap highlights the flexibility and comprehensiveness of parental selection for crossing to discover more QTGs, which may not be easily or efficiently achieved in other multi-parental populations. Because the effect of a target QTL is unpredictable and diverse, the PDI between minor-phenotypic-difference accessions for crossing should be diverse accordingly and must be controlled in a certain range, such as from 8% to 25%, which should not be either too big or too small in RapMap. To find more QTL genes, the parents selected for the construction of genetic populations should be flexible rather than fixed, unlike MAGIC and NAM. Thus, besides these requirements of constructing a series of F_2_GPs coupled with the consideration of known genes and the kinship between parents, we do not propose to specify other explicit requirements for parent selection, because this step or idea is finally guaranteed by the co-segregation standard (Fig. 1c).

RapMap also highlights the simplicity and multiple parents in the construction of multiple F_2_GPs. The construction of multiple F_2_GPs only requires a one-time cross between minor-phenotypic-difference parents followed by one subsequent self-pollination (Fig. 1a). No additional crossing and selfing is required for F_2_GPs, unlike the complex construction of advanced generation populations (RIL, MAGIC and NAM), which is particularly important for self-pollination species whose manual crossing is onerous^14^.

The map-based cloning approach employing recombinants has been proved as the most successful and reliable strategy to isolate the causal QTGs in crops^8–12^. During the last two decades, many qualitative- and quantitative- trait genes have been cloned using F_2_ populations. Besides the difficulty in obtaining a single-locus segregation line, the two major limitations of F_2_ populations are: (1) nearly half of genes in F_2_ lines are heterozygous, which may impede the full observation of their phenotypes in mapping; (2) only two alleles at a given locus segregate from bi-parents, which will lead to a limited number of QTL discovered in such populations. These are the two major reasons for the construction of RIL, DH, NAM and MAGIC populations to make all the loci homozygous in each line to fully address the phenotypic contribution of each allele, and try to employ multi-parental populations (GWAS, NAM and MAGIC) to detect more QTL. As a similar case in multi-parental populations, simply increasing population size in bi-parental populations can also increase mapping precision. There are at least six notable advantages of using F_2_GPs for QTL mapping and cloning in RapMap over these traditional populations. (1) RapMap only uses homozygous genotypes instead of heterozygous plants in F_2_ progenies to ensure the integration of the three-in-one step by the co-segregation standard. (2) NIL-LLs from enough F_2_ heterozygous plants can provide sufficient recombinant events in a target QTL for its fine mapping and cloning. (3) RapMap requires a series of F_2_GPs at similar or different bi-parent gradient levels, which may have good repeatability of genetic loci in diverse genetic backgrounds. (4) The genetic population that does not meet the co-segregation standard of single-locus genetic models could be replaced by a phenotype-well-segregating F_3_ or F_4_ family derived from a randomly selected low-, medium- or high-value F_2_ or F_3_ heterozygous line (Fig. 1f), which is difficult to be realized by RIL, DH, NAM, and MAGIC populations (usually with all the loci homozygous in each line). To clone a QTL, it is not always necessary to purify all the other genetic backgrounds by backcrossing. The only necessary thing is to ensure that the target locus is heterozygous as detected by phenotypic segregation and confirmed by the co-segregation standard, and other limited QTL are homozygous in segregating progenies. Thus, to clone a QTL from a bi-parent population with more than one segregating locus, we can dissect the multi-QTL segregating population into a one-QTL segregating population by selfing (rather than backcrossing) and selecting F_2_ or F_3_ progenies with good phenotypic segregation (Fig. 1f and the *GL7* gene from Cross 5 in Fig. 2). (5) Because the parental accessions can cover as much phenotypic diversity as possible across diverse germplasms, the resulting F_2_GPs will be the simplest and easiest option to achieve the goal of revealing the full genetic architecture of important traits. (6) RapMap is unbiased. In addition to major genes *GS3* and *GW5*, the heritability contribution of the other six genes is small or moderate in the natural population (Fig. 4a–c; Supplementary Data 1; Supplementary Table 1 and 2). So, we state that RapMap can clone not only major genes but also minor genes or rare alleles, and thus has no preference for identifying genes with various effects. Thus, multiple F_2_GPs in RapMap provide a high power to detect a QTL segregating in a bi-allele system, low population structure to control false positives, high genetic diversity to achieve multi-QTL mapping using multi-parental gradient populations, mapping QTL of small effect and low allele frequency, and more recombination events from NIL-LLs to reveal the causal genes, which are all favorable characteristics for both mapping and cloning of QTL.

RapMap is highlighted as a gene-cloning-orientated method. It should be noted that traditional QTL mapping, GWAS, MAGIC or NAM mainly offer preliminary mapping with high resolution for some loci, while the causal genes of a phenotype have been rarely identified^14^. To discover a causal gene using these populations, much additional time and cost have to be spent. Limited recombination coupled with many interference factors or loci from the background usually leads to a large QTL interval obtained using the traditional bi-parental methods^8–12^. For GWAS, the low LD decay, especially for the self-pollinated crops like rice, usually restricts the mapping resolution^15,16^. In theory, the design of multiple parental populations such as MAGIC and NAM can greatly solve this problem, but there are still many QTL intervals that are too large to be cloned directly^14^. The larger number of accumulated recombination events in F_2_, GWAS, MAGIC and NAM can increase the mapping resolution of QTL. In MAGIC populations of ~530 RILs, QTL for various traits were mapped to intervals of 0.3–6 Mb in *A. thaliana*^14,17^, and 1.5–17 Mb in maize^18^. In a rice MAGIC population of 1316 RILs, the mapping intervals were ~700 Kb on average^14,50^. However, it is still difficult to isolate most causal genes in such big regions without enough recombination events that need to be screened from thousands of individuals^14^. Moreover, it takes years to confirm the effect and then start the fine-mapping process of the QTL with the construction of NILs from many consecutive backcrosses or intermediate crosses performed during population development (Fig. 1e)^3–9^. So far, no gene has been identified by MAGIC and NAM populations in rice since the first MAGIC and NAM case of rice was reported in 2013 and 2017, respectively^12,14,50,51^. In RapMap, however, detecting a QTL, verifying its effect and obtaining its NIL-LLs, which are three decisive rate-limiting steps for cloning QTL commonly faced in other methods, are integrated into a three-in-one step ensured by the co-segregation standard (Fig. 1a–e). This advantage directly simplifies the common and necessary rate-limiting steps in isolating QTL, and greatly enhances the efficiency and accuracy of both QTL identification and gene discovery relative to traditional methods. Sufficient recombinants from the heterozygous lines of F_2_GP are critical for the map-based cloning of a QTL gene (Fig. 1c, d, Figs. 2–3). Thus, RapMap integrates the merits of multi-parent populations and the traditional map-based cloning approach, greatly promoting the precision and efficiency of isolating the causal genes, rather than being limited to the stages of primary mapping.

RapMap also integrates the merits of both the higher reliability of linkage analysis and the throughput of GWAS, MAGIC and NAM in mapping a QTL, which can be attributed to the flexible and comprehensive selection of target traits and parents as well as simplicity and rapidity of genetic population construction. This allows the rapid and systematic identification of as many QTGs as possible for a trait by constructing as many gradient populations as possible from diverse germplasms, while other methods are based on single bi-parental and specific multi-parental populations, and usually provide limited pieces in the complex jigsaw of a trait^7–14^. Thus, as a high-throughput mapping and cloning approach, RapMap holds tremendous promise for revealing the full genetic architecture of important traits in crops.

RapMap has a lower cost of time and labor to achieve the same advantages as multi-parental mapping populations including superior genetic diversity, smaller haplotype blocks and higher mapping power. For the traditional QTL mapping method, a large number of individuals and genome-wide genotyping of each individual are necessary to localize QTL in a single mapping trial^6^. After general QTL localization using the primary mapping populations, advanced generation backcross lines (NILs) for high-resolution mapping of QTL should be available. In RapMap, the flexibility of parent selection, the speed construction of multiple F_2_GPs, the three-in-one feature and the direct obtaining of NIL-LLs greatly reduce the time and labor cost of multiple QTL mapping and cloning as a whole. For RapMap, we can complete the primary mapping just by the BSA-based strategy without genome-wide genotyping of each individual in F_2_GPs, verify the true QTL by the co-segregation standard and discover the gene by fine-mapping using NIL-LLs. Multiple F_2_GPs make it possible to clone many different genes or alleles from diverse genetic backgrounds (Figs. 2 and 3), which can save the average cost for a single gene. RapMap allows researchers to investigate different traits using the same set of germplasms with reusable genotype and phenotype data. RapMap can flexibly select different parents to make crosses for one trait in one generation, without crossing for many years. Once a QTL is confirmed by the co-segregation test, it is easier for RapMap to enhance the QTL mapping resolution using abundant NIL-LLs and complete the gene discovery. Thus, there is no need to repeatedly phenotype the lines of F_2_GPs for verification. Technically, in addition to crossing, the only work needed in RapMap is the genotyping and phenotyping of recombinants screened from NIL-LLs. This important step of discovering the underlying genes is realized by an automatic high-throughput detection technology of KASP in a cost-effective way, which greatly simplifies the genotyping and reduces the time cost of final gene discovery. In summary, the design of RapMap integrates simple crossing and automatic genotyping technologies, and as an innovative strategy for high-throughput QTL mapping and cloning, it displays simple, fast, efficient and flexible characteristics.

RapMap can identify more QTL with much larger PVEs and smaller regions than the traditional QTL, GWAS, MAGIC and NAM methods in rice. Compared with these populations, RapMap can identify more QTL, which is determined by the inherent advantages of multiple F_2_GPs. Results from the three typical QTL mapping cases of rice grain size using RIL or F_2:3_ populations demonstrated that the number of QTL verified in RapMap is larger than the number of QTL clusters mapped using traditional methods^52–54^. Compared with two GWAS cases using the same ~533 samples in our study (Supplementary Fig. 10) and others^55^, and a GWAS case using even 3000 samples with sufficient statistical power^56^, RapMap is still superior to GWAS in the number of QTL detected, the mapping resolution and the PVE. In the very limited literature on rice QTL mapping by MAGIC populations (no report on grain-size QTL mapping using a NAM population in rice), Ayaad et al. identified only two grain-length and two grain-width QTL^51^, which is similar to the GWAS results using the same mini-core collection. It can be expected that if the number of crosses with high genetic diversity increases, the number of QTL identified by RapMap will continue to increase. Moreover, the QTL identified by RapMap have much higher PVE in each F_2_PG (Supplementary Table 1 and 2), which is determined by the co-segregation standard. The average PVEs of QTL co-localized with our study in the three typical works using the traditional mapping method were 7.3%^52^, 28.3%^53^, and 35.1%^54^, which are much lower than those (67.4%, 81% and 86%) by RapMap (Supplementary Table 1 and 2). The average PVEs of QTL identified by the GWAS and MAGIC population and co-localized with ours were <10%^55^ and <21.5%^51^, respectively, also much lower than those identified by RapMap (Supplementary Table 1 and 2). The main reason may be that the QTL in RapMap were verified by the co-segregation standard, and thus the F_2_GP is the single gene segregation population and the identified QTL is a major gene in the bi-parental population. The higher PVEs of QTL obtained in RapMap finally suggested that the method can not only capture the genes with the largest effect (*GS3* and *GW5*) but also discover the genes with rare variations and minor effects (*GS2* and *GL1*) at the population level, which failed to be identified by GWAS and MAGIC due to their low allele frequencies. All the peak SNPs of QTL detected by GWAS and MAGIC were 16–368 kb away from the corresponding known genes (Supplementary Fig. 10)^51,55,56^, and therefore it is hard to quickly realize the final cloning of these QTL.

The co-segregation standard that the target phenotypes of two homozygous genotypes of a QTL in progenies of any segregating population can be distinguished (Fig. 1c), is firstly raised as the necessary and sufficient condition of single-locus genetic models and also as the quality-control standard for the three-in-one framework featured in RapMap. The co-segregation standard of benchmarking QTL mapping is the cornerstone of RapMap, while the construction of multiple F_2_GPs to most probably meet the quality-control standard is its technical strategy. The co-segregation standard also helps traditional geneticists to solve the confusing problems often faced. How many times of backcrossing are needed to successfully construct NILs in traditional QTL cloning? How to determine whether a mapping population is controlled by a single-locus? Is it right that the control of a single-locus or multiple loci is determined by a bimodal (or 3:1) or normal distribution of progeny phenotypes, respectively? Hence, the co-segregation standard can also be used to improve the efficiency of traditional genetic analysis. In RapMap, what we should pay more attention to is the co-segregation standard. It is noteworthy that the co-segregation standard in RapMap is the major premise and the important nature of QTL cloning that has not been revealed and applied by researchers. The co-segregation standard coupled with multiple F_2_GPs ensures that RapMap can apply to organisms that are easy to crossbreed and reproduce.

Unlike other methods, RapMap is a solid phenotype- and genotype-based instead of software- or statistics-dependent method that may introduce false positives or false negatives. All the previously reported QTL mapping methods, such as F_2_, RIL, DH, GWAS, MAGIC, NAM, QTL-seq, QTG-seq, and MutMap, require complex software and/or statistical analyses, or significant effort to distinguish true QTL from false positives or false negatives. In RapMap, we only need to phenotype the individuals of each F_2_GP and recombinants from its NIL-LLs, and genotype the two DNA pools and the recombinants in a QTL from NIL-LLs, and then fine map the QTL by combining the above data to isolate the causal gene (Figs. 1–3). Thus, RapMap can directly link the phenotype of a QTL with its genotype without complex software or statistical analysis.

It is necessary to find minor-effect genes by RapMap and other alternative methods. The genetic contributions or PVEs of the eight genes to grain size/shape were all calculated at the natural population level (Fig. 4a–f), but not at the bi-parental population level (Supplementary Table 1 and 2). These are two different concepts with totally different PVEs. If the functional natural variation of a QTL is common and selected by domestication and improvement, it will be widely distributed in a natural population and contribute more to phenotype variation (such as *GS3* and *GW5*). If the natural variation of a QTL is rare and not under strong selection, it will show a small effect in a natural population due to the low allele frequency (such as *GS2* and *GL1*). However, it shows a much greater effect in bi-parental populations, especially in F_2_GPs (Supplementary Table 1 and 2). For example, the grain length variation explained by *GS2* is 83% in F_2_GP of Cross 8 and 72% in an F_2_ population reported by Hu et al.^39^, although it is 3% at the population level in this study. Moreover, the discovery of small-effect or low-frequency genes by RapMap and other methods provides more gene resources with great potential to facilitate sustainability for crop breeding and improvement^3,8–10,12^. The introgression of the rare *GS2*^*BDL*^ allele into the current high-yield cultivar 9311 in China could significantly enhance grain weight by 40.3% and increase grain yield per plot by 14.3%^39,40^. Both major and minor genes are needed to provide flexibility in molecular design breeding for desired traits in crops. Because the genes with the major effect at a population level were often or first selected and fixed into the genetic background of elite cultivars, there may be no extra space for further improvement by themselves. Although some rare variations (such as *GS2*) or minor-effect genes (such as *GL1* or *GW5.1*) contribute little to the PVE at a population level, they could also be important gene resources for the sustainable breeding improvement of crops. With continuous introgression, the minor genes will be of more value in the breeding population where their frequencies can be increased for the benefit of future production. Furthermore, many more QTGs with both major and minor effects for a specific trait need to be identified to reveal the regulatory mechanism of QTG interactions underlying natural variation and genetic diversity, which is an important aim and also a big challenge in the crop science community^4,7,8,11,21^.

Our study implied that two-thirds of the genetic variations for grain size/shape have been dissected by RapMap at the population level, and nearly one-third of the phenotypic effects remain elusive (Fig. 4a–f). The missing heritability of an extra 30% may result from several reasons. (1) Due to the limited sample size or some non-representative parents selected in RapMap, other genetic effects or rare variants cannot be fully detected. Another round of RapMap by selecting new gradient accessions will be needed to find other QTGs. (2) Epigenetic modifications and structural variants are candidates to explain a part of the missing heritability that is not effectively captured by usual genotyping from sequencing or SNP chip^57–59^. We generally score heritability using variations of DNA sequence, but variations in DNA methylation or other modifications and DNA structures (not easily tagged by usual genotyping) are prevalent and can also cause phenotypic variation. (3) Extra heritability may be due to genetic interactions (epistasis), environmental effects and experimental errors from large-scale phenotyping^4,7,8^. The above reasons may explain why an extra 30% heritability was not found in RapMap, and also direct our future research.

In this study, RapMap could simultaneously identify eight grain-size genes in three years, including six known genes (*GS3*, *GL7*, *GS2*, *GW5*, *GW8,* and *GW7*), two unreported *GS3* alleles (*GS3-5* and *GS3-6*) and two unknown genes (*GL1* and *GW5.1*), which include both major effects and minor effects on grain size in natural germplasms, while it would take about 8–10 years under regular programs (Figs. 1–4). Using recombinants to fine map a QTL in map-based cloning programs and even in other mapping methods is the most efficient strategy to identify a causal gene underlying the QTL^8–14^. However, it is sometimes extremely difficult to narrow a QTL down to a very small chromosomal region with few genes, due to the QTL effect, population size, available molecular markers and recombination frequency, such as the cases of some grain-width QTL in this study (Fig. 3). To cope with these problems, many candidate gene strategies could be used to accelerate the gene discovery in a large fine-mapping QTL region, including (1) finding functional variations (InDels, stop-codons or other large variations resulting in changes of RNA level or protein functions) by comparative sequencing between bi-parents, (2) checking homologous genes or proteins with similar functions in other species or function-related pathways, (3) discovering differential expression genes between two NILs, parents or haplotypes, (4) checking target tissue-specific or -abundant expression genes or excluding non-expression genes in target tissues, and (5) probing candidate genes using good gene annotation, mutants, cDNAs or ESTs as important resources. Complementation transformation of a functional or strong allele driven by its native promoter into a parent or NIL with a null or weak allele is the standard for declaring the isolation of a QTG that controls a natural variation trait, just like the function confirmation of the two genes (*GL1* and *GW5.1*) in this study (Figs. 2i and 3i).

The domestication signatures of grain-size variation at a population level were revealed through in-depth analysis of the eight genes with a large and geographically diverse population (Figs. 4 and 5). Directional selection is the shift of the mean value of a trait from one position in a frequency histogram to a distinct value^26^. We observed the directional accumulation of slender- and long-grain alleles of the eight grain-size genes during the rice improvement process from landrace to cultivar in *indica* but not in *japonica* rice, which resulted in a significant shift of grains to a slenderer and longer shape. Differential geographical distribution and ecological habits may be one of the reasons for the directional selection difference between *indica* and *japonica* populations (Supplementary Fig. 6–8). Usually, slender- and long- grains in rice with aroma are considered to have superior quality over short- and bold- grains^60^. These preference tendencies may be attributed to the meeting of human requirements such as taste, yield, quality and cultivation practices. For example, slender *indica* grains always have excellent appearance, cooking and eating quality with much lower grain chalkiness than wide grains^60^. Larger or longer seeds were selected to increase grain yield and produce more food during early domestication. An increase in grain size is an early adaptive response to human cultivation, and greater grain size is strongly correlated with larger seedlings in many cereal and legume species^26^. Therefore, human cultivation practices coupled with food preferences might contribute to the main tendency of directional selection. The allele frequency and haplotype analyses provide more opportunities for genomic breeding of ideal cultivars with desired grain shapes.

Selection sweeps of eight grain-size genes were identified in modern cultivars, landraces and wild rice, providing additional evidence for the strong selection pressure on these genes and the selection signatures shaped in the early domestication by ancient humans (Fig. 5 and Supplementary Fig. 9). Artificial selection on target traits based on requirements and cultivation practices of modern humans was performed in the past. As a result, the alleles with major effects on the target traits have been strongly and correspondingly selected. The coincidence of large grain-size effects of *GW5*, *GS3,* and *GL7* and strong selection pressure before the completion of rice domestication may help to clarify the relationship between them. As we expected, the genetic effects of the eight grain-size genes were found to be highly and positively correlated with the selection strength and nucleotide variation intensity, which is different from that reported in the previous studies^61^. Selection strength on the eight genes plays an essential role in changing the correlation between accumulation of DNA variations and grain-size phenotypes in the large diverse population. These conclusions may be general to other traits, though more data are needed to verify its power and robustness further. Crop domestication is a lengthy process of selection for variation in a suite of traits that eventually makes a plant cultivable^62,63^. Mimicking this process through neo-domestication of wild, landrace or cultivar species using multiple QTGs coupled with gene editing technologies may be an alternative way to breed modern cultivars quickly^63^.

In this study, we have developed RapMap, a practical and versatile approach for the rapid discovery of functional genes in a high throughput pattern, which was successfully applied to the cloning of grain-size genes in rice. Although the concept of RapMap was well demonstrated firstly in rice, RapMap could be immediately extended to any other crops that are easy to crossbreed and reproduce. Many crop germplasm collections^3,8,10,12,15,63^, which are recently available for rice, maize, wheat, barley, millet, sorghum, soybean, and pea crops and their wild relatives, will facilitate the immediate and comprehensive application of RapMap. The great ability of rapidly cloning agriculturally valuable genes by RapMap will further increase the value of these germplasm collections. Overall, we believe that RapMap may become a classical paradigm and benchmark for identifying QTGs in functional genomics owing to its great potential and notable advantages. Various functional genes from diverse germplasms could be fully applied for accelerating the genetic improvement of valuable traits by precise genomic breeding in plants.

## Methods

### Rice mini-core collection

The rice parental lines for the generation of F_2_ gradient populations were selected from a diverse mini-core collection including 541 *O. sativa* accessions^29,30^. The mini-core collection contained both landraces and some elite varieties, including 215 varieties from China’s core collection of *Oryza sativa* L. and 326 varieties from other 58 countries worldwide (Supplementary Data 1). All the accessions were grown in the normal season at the Experimental Stations of Huazhong Agricultural University, Wuhan and Lingshui, China, with a space of 16.5 cm between rows and 26 cm between plants. The cultivation followed normal field management such as irrigation, fertilizer application and pest control.

### Evaluation of grain size and other yield traits

Seed size (grain length and width) and other yield traits were measured as described by Li et al.^64^. Harvested rice grains were air-dried and stored at room temperature for at least one month before measuring. Fully filled grains were used to measure the grain length, width and weight. Ten randomly chosen grains from each plant were lined up lengthwise along a vernier caliper to measure grain length and arranged widthwise to measure grain width.

### Prediction of the minimum number of crosses for the construction of F_2_ gradient populations

The minimum number of crosses for the Construction of F_2_ gradient populations could be predicted by Eq. 1.

1 $$N=PD{I}_{(corecollection)}/PD{I}_{(threshold)}$$

where *N* is the minimum number of crosses needed; *PDI*_(core collection)_ can be obtained by dividing the minimum by the difference of maximum and minimum of the trait values in the core collection; the *PDI*_(threshold)_ is 20% as mentioned above. Taking grain size in this study as an example, the number of crosses for grain length and width is *N*_(grain length)_ = (9.27−3.97)/3.97/20% = 6.68 ≈ 7 and *N*_(grain width)_ = (3.37−1.66)/1.66/20% =5.15 ≈ 6, respectively.

### Construction of F_2_ gradient populations and field trials

According to the phenotype values of the collection, we selected 12 and 10 accessions with gradient phenotypes of grain length and width, respectively (Figs. 2a and 4a, Supplementary Data 1). The parental materials of the F_2_GPs used in this study are listed in Supplementary Data 1. Of all the parental materials, Chuan 7, Zhenshan 97, Peiai 64 S, 9311, Chenghui 3203, WZ1, Chuan 106B, SYB6, Domsiah, ZIRI, Minghui 63, and B805D belong to *indica* subspecies, while Heen GW, Zhimali, SYB5, Iksan438 and Nipponbare belong to *japonica* subspecies. Then, we arranged the minor-phenotypic-difference accessions as crossing parents to generate F_1_ seeds in March 2016 at Hainan. The F_1_ plants from each cross were independently self-pollinated in July 2016 at Wuhan, and each F_2_GP (~200 plants) from each F_1_ was grown independently in the field of Hainan in April 2017 for measuring their target phenotypes after harvesting. All the field management of F_2_GPs was the same as that of the mini-core collection.

### Genotyping of bulked DNAs by the RICE6K array and high-resolution sequencing

Seeds of F_2_ plants with extreme phenotypic values were used for bulking. For each bulk with extreme phenotypes, more than 25 plants and 10 seeds per plant were selected. Seeds of each bulk were grown in a culture room with an average day and night temperatures of 30 and 25 °C, respectively, and 75% relative humidity under a 12 h light and 12 h dark cycle. The leaves of 10-day-old seedlings were equally mixed for bulking. DNA extraction of bulked leaves was performed using the cetyltrimethylammonium bromide (CTAB) method. The RICE6K hybridization, scanning and the following further analyses were performed by China National Seed Group Co., Ltd^31^.

DNA deep sequencing of four samples (two DNA pools from Cross 4 for grain length in Fig. 2 and two DNA pools from Cross 7 for grain width in Fig. 3) was performed by Annoroad Gene Technology Co., Ltd (Beijing, China) using Illumina Hiseq X Ten platform in a 2 × 150 pair-ended manner. After the quality-control, short reads in which more than 10% of sequenced nucleotides exhibited a Phred quality score of <30 were excluded from the following analysis. The genotyping and primary mapping procedures were as described by Takagi et al.^32^.

### Design of InDel and SNP markers for verification and fine mapping of QTL

After analysis of the QTL based on RICE6K or NGS, the potential QTL region was available. Based on the RiceVarMap database covering most parental materials in our study (http://ricevarmap2.ncpgr.cn/v1/)^33^, we could effectively find the insertion-deletion (InDel) polymorphic variations between bi-parents and rapidly design InDel markers in a candidate interval. SNP markers gained by sequencing may be needed for further fine mapping when InDel markers are not available in a small region. All the marker primers are listed in Supplementary Data 2.

### Rapid DNA extraction technique

For the verification of QTL and identification of recombinants, we used an ultra-fast protocol for rapid DNA extraction as follows^65^. One-centimeter leaf blade was cut off and placed into a 96-well deep-hole plate. Samples were ground with 200 μL of NaOH (0.3 M) for each well, centrifuged for 1 min at 800 × g and boiled for 45 s. Then, the samples were mixed with 300 μL Tris-HCl (0.75 M, pH7.4–7.8), boiled for 45 s and centrifuged for 1 min at 800 × g. After that, 5 μL of the supernatants and 45 μL ddH_2_O were transferred to the 96-well PCR plate for screening of recombinants.

### Screening of recombinants by KASP

KASP technology was employed to genotype the potential recombinants in each QTL region^34^. For each QTL, the flanking markers were designed using the ‘SNP Primer’ tool (http://www.snpway.com/snpprimer/) based on the variations of SNPs retrieved from the RiceVarMap database^33^. Furthermore, the screening of recombinants was conducted using the KASP primers by genotyping about 5000 individuals of an F_2:3_ population developed from F_2_ plants with heterozygous genotypes of a target QTL. The plants with different genotypes at two markers covering the QTL interval were picked as recombinants, whose phenotypes were tested in the field for determining their corresponding genotypes of the target gene. All the KASP primers are listed in Supplementary Data 2.

### RNA isolation and RT-PCR analysis

Total RNA was extracted from young panicles using the kit (TransZol, TransGen Biotech, Cat. No.ET101-01). The extracted RNA was incubated with RNase-free DNase I (Amplification Grade, Invitrogen, Cat. No.18068-015) to eliminate genomic DNA contamination. The first-strand cDNA was synthesized from 2 µg total RNA using the M-MLV reverse transcriptase (Invitrogen, Cat. No.28025013). All the experiments were conducted according to the manufacturer’s instructions with at least three biological replicates. Semi-quantitative reverse transcription-polymerase chain reaction (RT-PCR) was performed to determine the expression level of the target gene. The rice *Ubiquitin* gene (LOC_Os03g13170) was used as an internal control. All the RT-PCR primers are listed in Supplementary Data 2.

### Construction and genetic transformation of CRISPR/Cas9 and complementation vectors

CRISPR/Cas9 technology was used to generate gene-edited plants of target genes^66^. For a single-target vector, the gene-specific guide sequence (sgRNA) was designed and integrated into the *OsU6* promoter by an overlapping PCR. Then, the sgRNA transcriptional unit was inserted into the *pCXUN-CAS9* plasmid at the *Kpn*I site through Gibson assembly^67^. For a double-target vector, the second sgRNA was designed and integrated into the *OsU3* promoter by an overlapping PCR. Subsequently, the second sgRNA transcriptional unit was inserted into the obtained single-target vector at the *Sac*I site through Gibson assembly. The CRISPR constructs of *GW5.1* with a single target and *GL1* with double targets were transformed into *Agrobacterium tumefaciens* strain EHA105 and then introduced into Zhonghua 11 by *Agrobacterium*-mediated transformation^64^.

To validate the function of *GW5.1* on grain width, a 6 kb genomic fragment containing a 3.5 kb fragment upstream of *GW5.1* and the coding sequence was amplified from Iksan438 genomic DNA and inserted into the plant binary vector pCAMBIA 1301-Flag between the *EcoR*I and *Kpn*I sites using Gibson assembly. After the destination construct was verified by sequencing, it was introduced into loss-of-function ‘AA’ callus by *Agrobacterium*-mediated transformation^64^. To validate the function of *GL1* on grain length, a 5.5 kb genomic fragment containing a 2.3 kb fragment upstream of *GL1* and the coding sequence was amplified from WZ1 genomic DNA and inserted into the pCAMBIA 1301-Flag vector. Then, the construct with the right sequence was introduced into ZH11. All the transgenic plants were grown in the transgenic paddy fields of the Experimental Stations of Huazhong Agricultural University, Wuhan and Lingshui, China.

All the primers used in the genetic transformation are listed in Supplementary Data 2.

### Dual-LUC analysis

For the promoter and 3′UTR activity analysis, the 2.3 kb promoter and 3’UTR fragments of *GL1* were amplified from WZ1, 9311, and ZH11, respectively, and cloned into pGreenII 0800-LUC reporter vector^68^ with the promoter and 3′UTR fragment flanking the *LUC* CDS. In the same construct, a *REN* gene under the control of a CaMV 35 S promoter that provided an estimate of the strength of transient expression was used as an internal transformation control. Each of the promoter-LUC-3′UTR fusion constructs was used for the transient transformation in rice protoplasts. All the primers used in the Dual-LUC analysis are listed in Supplementary Data 2.

The activity of LUC and REN was assayed using the Dual-Luciferase Reporter Assay System (E1910, Promega)^68^. After transient transformation, protoplasts were incubated for 16 h at 25 °C and the harvested protoplasts were lysed in 60 μl of Passive Lysis Buffer. 30 μl of this crude extract was assayed in 30 μl of Luciferase Assay Buffer mixed with Substrate, and the chemiluminescence was measured using the TECAN SPARK Microplate Reader with a 10 s delay. 30 μl of Stop and Glow™ Buffer mixed with the substrate was then added and a second chemiluminescence measurement was made with a 10 s delay. The ratio of LUC to REN activity was used as the relative promoter and 3′UTR activity for each allele. Three biological replicates were assayed per construct.

### Evaluation of genetic effects of the eight grain-size genes

Multiple linear regressions were performed to estimate the genetic effects of each gene and to predict the phenotype performance by using the function *lm()* in R (Version 3.6.2). The model was as follows:

2 $$Y={g}_{0+}{g}_{1}Marker1+{g}_{2}Marker2+\ldots +{g}_{i}Markeri+e$$

where *Y*, *g*_*0*_, *g*_*j*_ (*j* = 1, 2, …, i), *Marker*, and *e* represent the phenotype, intercept, regression coefficient, genotype of the functional marker from a gene, and random error, respectively. 10-fold cross-validation was conducted to assess the model prediction performance. The relative importance of each variable (allele) for the phenotype explanation was estimated by calculating the relative weight. The 10-fold cross-validation and the estimation of relative importance were executed by using the R script. For the comparison of allelic combinations, one-way ANOVA was performed first and then the multiple comparisons were conducted by the least significant difference (LSD) method.

### Population genetic analysis of the eight grain-size genes

The genotypes of 446 rice wild species *Oryza rufipogon* were retrieved from Huang et al.^46^. The genotypes of 4726 rice landrace and cultivar were downloaded from RiceVarMap2 (http://ricevarmap.ncpgr.cn/v2/)^33^. The inference for landrace and cultivar was based on the plant height, the representative functional marker of the *SD1* gene and the known information and curated manually^32^. The nucleotide diversity level per silent-site was estimated as π by VCFtools^69^. We also checked the nucleotide diversity of a 2 Mb flanking genomic region of each gene with a 100 kb sliding window and a 20 kb step. The silent-site nucleotide diversity between accessions with long- and short-grain alleles or wide- and slender-grain alleles of each grain-size gene and each other gene within its surrounding 500 kb regions was compared for scanning the selective sweep valley. The selection pressure of each gene was the average ratio of nucleotide diversity of cultivars and landraces. The DNA variations of genes were measured by the Kimura 2-parameter which was calculated by MEGA X^70^ after comparing the gene sequences of the cultivars and the wild species *Oryza rufipogon* W1943. A genome-wide association study was conducted using the mini-core collection (Supplementary Fig. 10) by FaST-LMM software^71^ with genetic similarities used to estimate random effects. The phenotype association significance of the length-width ratio used in the correlation analysis was the minus log-transformed *P*-value.

### Reporting summary

Further information on research design is available in the Nature Research Reporting Summary linked to this article.

## Supplementary information

Supplementary information

Supplementary Data 1

Supplementary Data 2

Supplementary Data 3

Supplementary Data 4

Description of additional supplementary files

Reporting Summary

## Data Availability

The data supporting the findings of this work are available within the paper and its Supplementary Information files. A reporting summary for this article is available as a Supplementary Information file. The SNP data of the mini-core collection can be retrieved from http://ricevarmap.ncpgr.cn/download/. Accession codes of all genes or alleles reported in the study are available in GenBank: cDNA of *GS3-5* in Zhimali and Heen GW, MW808603; cDNA of *GS3-6* in SYB5, MW808604; genomic DNA of *GL1* in WZ1, MW804343; genomic DNA of *GL1* in 9311, MW824660; genomic DNA of *GL1* in Zhonghua 11, MW824661; genomic DNA of *GW5.1* in Ikasan438, MW800744. DNA deep sequencing for two DNA pools from Cross 4 for grain length and two DNA pools from Cross 7 for grain width are deposited in the SRA database of NCBI with accession PRJNA761681 and PRJNA761576, respectively. Source data are provided with this paper.

## Source data

Source Data

## References

[1] Stange M, Barrett R, Hendry A (2021). The importance of genomic variation for biodiversity, ecosystems and people. Nat. Rev. Genet..

[2] Rheenen W, Peyrot W, Schork A, Lee S, Wray N (2019). Genetic correlations of polygenic disease traits: from theory to practice. Nat. Rev. Genet..

[3] Bazakos C (2017). New strategies and tools in quantitative genetics: how to go from the phenotype to the genotype. Annu. Rev. Plant Biol..

[4] Jakobson C, Jarosz D (2020). What has a century of quantitative genetics taught us about nature’s genetic tool kit?. Annu. Rev. Genet..

[5] Boyle E, Li Y, Pritchard J (2017). An expanded view of complex traits: from polygenic to omnigenic. Cell.

[6] Mackay T, Stone E, Ayroles J (2009). The genetics of quantitative traits: challenges and prospects. Nat. Rev. Genet..

[7] Costanzo M (2019). Global genetic networks and the genotype-to-phenotype relationship. Cell.

[8] Liang Y, Liu H, Yan J, Tian F (2021). Natural variation in crops: realized understanding, continuing promise. Annu. Rev. Plant Biol..

[9] Holland J (2007). Genetic architecture of complex traits in plants. Curr. Opin. Plant Biol..

[10] Wallace J, Rodgers-Melnick E, Buckler E (2018). On the road to breeding 4.0: unraveling the good, the bad, and the boring of crop quantitative genomics. Annu. Rev. Genet..

[11] Benfey P, Mitchell-Olds T (2008). From genotype to phenotype: systems biology meets natural variation. Science.

[12] Wei X (2021). A quantitative genomics map of rice provides genetic insights and guides breeding. Nat. Genet..

[13] Yamamoto T, Yonemaru J, Yano M (2009). Towards the understanding of complex traits in rice: substantially or superficially?. DNA Res..

[14] Scott M (2020). Multi-parent populations in crops: a toolbox integrating genomics and genetic mapping with breeding. Heredity.

[15] Huang X, Han B (2014). Natural variations and genome-wide association studies in crop plants. Annu. Rev. Plant Biol..

[16] Xiao Y (2017). Genome-wide association studies in maize: praise and stargaze. Mol. Plant.

[17] Kover P (2009). A multiparent advanced generation inter-cross to fine-map quantitative traits in *Arabidopsis* thaliana. PLoS Genet..

[18] Dell’Acqua M (2015). Genetic properties of the MAGIC maize population: a new platform for high definition QTL mapping in *Zea mays*. Genome Biol..

[19] Gage J, Monier B, Giri A, Buckler E (2020). Ten years of the maize nested association mapping population: impact, limitations, and future directions. Plant Cell.

[20] Li N, Xu R, Li Y (2019). Molecular networks of seed size control in plants. Annu. Rev. Plant Biol..

[21] Fan Y, Li Y (2019). Molecular, cellular and Yin-Yang regulation of grain size and number in rice. Mol. Breed..

[22] Zuo J, Li J (2014). Molecular genetic dissection of quantitative trait loci regulating rice grain size. Annu Rev. Genet.

[23] Klingenberg C (2010). Evolution and development of shape: integrating quantitative approaches. Nat. Rev. Genet..

[24] Hufford M (2019). Crop biodiversity: an unfinished magnum opus of nature. Annu. Rev. Plant Biol..

[25] Olsen K, Wendel J (2013). A bountiful harvest: genomic insights into crop domestication phenotypes. Annu. Rev. Plant Biol..

[26] Purugganan M, Fuller D (2009). The nature of selection during plant domestication. Nature.

[27] Purugganan MD (2019). Evolutionary insights into the nature of plant domestication. Curr. Biol..

[28] Zou C, Wang P, Xu Y (2016). Bulked sample analysis in genetics, genomics and crop improvement. Plant Biotechnol. J..

[29] Xie W (2015). Breeding signatures of rice improvement revealed by a genomic variation map from a large germplasm collection. Proc. Natl Acad. Sci. USA.

[30] Chen W (2014). Genome-wide association analyses provide genetic and biochemical insights into natural variation in rice metabolism. Nat. Genet..

[31] Yu H (2014). A whole-genome SNP array (RICE6K) for genomic breeding in rice. Plant Biotechnol. J..

[32] Takagi H (2013). QTL-seq: rapid mapping of quantitative trait loci in rice by whole genome resequencing of DNA from two bulked populations. Plant J..

[33] Zhao H (2015). RiceVarMap: a comprehensive database of rice genomic variations. Nucleic Acids Res..

[34] Semagn K (2014). Single nucleotide polymorphism genotyping using Kompetitive Allele Specific PCR (KASP): overview of the technology and its application in crop improvement. Mol. Breed..

[35] Fan C (2006). *GS3*, a major QTL for grain length and weight and minor QTL for grain width and thickness in rice, encodes a putative transmembrane protein. Theor. Appl. Genet..

[36] Takano-Kai N (2009). Evolutionary history of *GS3*, a gene conferring grain length in rice. Genetics.

[37] Mao H (2010). Linking differential domain functions of the GS3 protein to natural variation of grain size in rice. Proc. Natl Acad. Sci. USA.

[38] Wang Y (2015). Copy number variation at the *GL7* locus contributes to grain size diversity in rice. Nat. Genet..

[39] Hu J (2015). A rare allele of *GS2* enhances grain size and grain yield in rice. Mol. Plant.

[40] Duan P (2015). Regulation of *OsGRF4* by OsmiR396 controls grain size and yield in rice. Nat. Plants.

[41] Wang S (2015). The *OsSPL16*-*GW7* regulatory module determines grain shape and simultaneously improves rice yield and grain quality. Nat. Genet..

[42] Wang S (2012). Control of grain size, shape and quality by *OsSPL16* in rice. Nat. Genet..

[43] Shomura A (2008). Deletion in a gene associated with grain size increased yields during rice domestication. Nat. Genet..

[44] Duan P (2017). Natural variation in the promoter of *GSE5* contributes to grain size diversity in rice. Mol. Plant.

[45] Liu J (2017). *GW5* acts in the brassinosteroid signalling pathway to regulate grain width and weight in rice. Nat. Plants.

[46] Huang X (2012). A map of rice genome variation reveals the origin of cultivated rice. Nature.

[47] Abe A (2012). Genome sequencing reveals agronomically important loci in rice using MutMap. Nat. Biotechnol..

[48] Zhang H (2019). QTG-Seq accelerates QTL fine mapping through QTL partitioning and whole-genome sequencing of bulked segregant samples. Mol. Plant.

[49] Wang C (2019). Dissecting a heterotic gene through GradedPool-Seq mapping informs a rice-improvement strategy. Nat. Commun..

[50] Raghavan C (2017). Approaches in characterizing genetic structure and mapping in a rice multiparental population. G3 Genes Genomes Genet..

[51] Ayaad M (2021). Bin-based genome-wide association studies reveal superior alleles for improvement of appearance quality using a 4-way MAGIC population in rice. J. Adv. Res..

[52] Zhao D (2017). Genetic dissection of large grain shape in rice cultivar ‘Nanyangzhan’ and validation of a grain thickness QTL (*qGT3.1*) and a grain length QTL (*qGL3.4*). Mol. Breed..

[53] Bai X (2010). Genetic dissection of rice grain shape using a recombinant inbred line population derived from two contrasting parents and fine mapping a pleiotropic quantitative trait locus *qGL7*. BMC Genet..

[54] Tan Y (2000). Genetic bases of appearance quality of rice grains in Shanyou 63, an elite rice hybrid. Theor. Appl. Genet..

[55] Yang W (2014). Combining high-throughput phenotyping and genome-wide association studies to reveal natural genetic variation in rice. Nat. Commun..

[56] Wang W (2018). Genomic variation in 3,010 diverse accessions of Asian cultivated rice. Nature.

[57] Xu J (2019). Population-level analysis reveals the widespread occurrence and phenotypic consequence of DNA methylation variation not tagged by genetic variation in maize. Genome Biol..

[58] Manolio T (2009). Finding the missing heritability of complex diseases. Nature.

[59] Kover P, Mott R (2012). Mapping the genetic basis of ecologically and evolutionarily relevant traits in *Arabidopsis thaliana*. Curr. Opin. Plant Biol..

[60] Fitzgerald M, McCouch S, Hall R (2009). Not just a grain of rice: the quest for quality. Trends Plant Sci..

[61] Lu L (2013). Natural variation and artificial selection in four genes determine grain shape in rice. New. Phytol..

[62] Meyer RS, Purugganan MD (2013). Evolution of crop species: genetics of domestication and diversification. Nat. Rev. Genet..

[63] Hickey L (2019). Breeding crops to feed 10 billion. Nat. Biotechnol..

[64] Li Y (2011). Natural variation in *GS5* plays an important role in regulating grain size and yield in rice. Nat. Genet..

[65] Lu J (2020). A direct PCR–based SNP marker-assisted selection system (D-MAS) for different crops. Mol. Breed..

[66] He Y (2017). Self-cleaving ribozymes enable the production of guide RNAs from unlimited choices of promoters for CRISPR/Cas9 mediated genome editing. J. Genet. Genomics.

[67] Gibson D (2009). Enzymatic assembly of DNA molecules up to several hundred kilobases. Nat. Methods.

[68] Hellens RP (2005). Transient expression vectors for functional genomics, quantification of promoter activity and RNA silencing in plants. Plant Methods.

[69] Danecek P (2011). The variant call format and VCFtools. Bioinformatics.

[70] Kumar S, Stecher G, Li M, Knyaz C, Tamura K (2018). MEGA X: molecular evolutionary genetics analysis across computing platforms. Mol. Biol. Evol..

[71] Lippert C (2011). FaST linear mixed models for genome-wide association studies. Nat. Methods.

