# Supplementary material for: The identification of grain size genes by RapMap reveals directional selection during rice domestication
Source: Nat Commun. 2021 Sep 28;12:5673. doi: 10.1038/s41467-021-25961-1 (PMC8478914; doi:10.1038/s41467-021-25961-1)
Supplement: Supplementary file 1 — Supplementary information [file 41467_2021_25961_MOESM1_ESM.pdf]

The identification of grain size genes by RapMap reveals directional selection during rice domestication

Zhang *et al.*

Table 1. Phenotypic variation explained (PVE) of eight grain-length QTL in each F_2GP and the mini-core collection estimated by linear regression.

	Grain length QTL in F_2GP			Grain length QTL in mini-core collection		
**Crosses**	**Variable**	**Estimate**	**Significance**	**Variable**	**Estimate**	**Significance**
Cross 1	Intercept	6.05	< 2.00e-16	Intercept	6.01	< 2.00e-16
	GS3 (B)	-0.57	1.46e-07	*GS3* (+)	1.29	< 2.00e-16
	Adjusted R^2	44%		Adjusted R^2	63%	
	P value	1.46e-07		*P* value	< 2.00e-16	
Cross 2	Intercept	7.28	< 2.00e-16	Intercept	6.01	< 2.00e-16
	GS3 (B)	-1.36	< 2.00e-16	*GS3* (+)	1.29	< 2.00e-16
	Adjusted R^2	85%		Adjusted R^2	63%	
	P value	< 2.20e-16		*P* value	< 2.00e-16	
Cross 3	Intercept	7.95	<2.00e-16	Intercept	6.01	< 2.00e-16
	GS3 (B)	-1.57	<2.00e-16	*GS3* (+)	1.29	< 2.00e-16
	Adjusted R^2	90%		Adjusted R^2	63%	
	P value	< 2.20e-16		*P* value	< 2.00e-16	
Cross 4	Intercept	10.06	<2.00e-16	Intercept	6.01	< 2.00e-16
	GS3 (B)	-1.13	<2.00e-16	*GS3* (+)	1.29	< 2.00e-16
	Adjusted R^2	79%		Adjusted R^2	63%	
	P value	< 2.20e-16		*P* value	< 2.00e-16	
Cross 5	Intercept	10.44	<2.00e-16	Intercept	6.25	< 2.00e-16
	GL7 (B)	-0.81	<2.00e-16	*GL7* (+)	0.91	< 2.00e-16
	Adjusted R^2	94%		Adjusted R^2	15%	
	P value	< 2.20e-16		*P* value	< 2.00e-16	
Cross 6	Intercept	10.85	< 2.00e-16	Intercept	6.21	< 2.00e-16
	GL1 (B)	-0.74	1.10e-15	*GL1* (+)	0.2	1.03e-2
	Adjusted R^2	50%		Adjusted R^2	1%	
	P value	< 2.20e-16		*P* value	0.0103	
Cross 7	Intercept	11.96	< 2.00e-16	Intercept	6.25	< 2.00e-16
	GL7 (B)	-1.03	3.13e-14	*GL7* (+)	0.91	< 2.00e-16
	Adjusted R^2	58%		Adjusted R^2	15%	
	P value	3.13E-14		*P* value	< 2.00e-16	
Cross 8	Intercept	14.59	< 2.00e-16	Intercept	6.29	< 2.00e-16
	GS2 (B)	-2.74	< 2.00e-16	*GS2* (+)	0.4	9.00e-05
	Adjusted R^2	83%		Adjusted R^2	3%	
	P value	< 2.20e-16		*P* value	9.00e-5	

'B' and '+' in parentheses of the 'Variable' column indicate the low-value genotypes of related genes in F_2GPs and high-value genotypes of related genes in the mini-core collection, respectively.

Table 2. Phenotypic variation explained (PVE) of seven grain-width QTL in each F_2GP and the mini-core collection estimated by linear regression.

	Grain width QTL in F_2GP			Grain width QTL in mini-core collection		
**Crosses**	**Variable**	**Estimate**	**Significance**	**Variable**	**Estimate**	**Significance**
Cross 1	Intercept	2.61	< 2.00e-16	Intercept	2.52	< 2.00e-16
	GW8 (B)	-0.27	< 1.02e-13	*GW8* (+)	0.33	< 2.00e-16
	Adjusted R^2	61%		Adjusted R^2	24%	
	P value	1.02E-13		*P* value	< 2.20e-16	
Cross 2	Intercept	2.77	< 2.00e-16	Intercept	2.52	< 2.00e-16
	GW8 (B)	-0.31	1.91e-13	*GW8* (+)	0.33	< 2.00e-16
	Adjusted R^2	51%		Adjusted R^2	24%	
	P value	1.91E-13		*P* value	< 2.20e-16	
Cross 3	Intercept	2.7	< 2.00e-16	Intercept	2.52	< 2.00e-16
	GW8 (B)	-0.32	2.42e-13	*GW8* (+)	0.33	< 2.00e-16
	Adjusted R^2	58%		Adjusted R^2	24%	
	P value	2.42E-13		*P* value	< 2.20e-16	
Cross 4	Intercept	2.82	<2.00e-16	Intercept	2.54	< 2.00e-16
	GW7 (B)	-0.23	<2.00e-16	*GW7* (+)	0.29	< 2.00e-16
	Adjusted R^2	90%		Adjusted R^2	17%	
	P value	< 2.2e-16		*P* value	< 2.20e-16	
Cross 5	Intercept	2.99	<2.00e-16	Intercept	2.52	< 2.00e-16
	GW5.1 (B)	-0.3	3.21e-11	*GW5.1* (+)	0.21	5.87e-12
	Adjusted R^2	60%		Adjusted R^2	12%	
	P value	3.21E-11		*P* value	5.87e-12	
Cross 6	Intercept	3.08	<2.00e-16	Intercept	2.38	< 2.00e-16
	GW5 (B)	-0.28	1.86e-10	*GW5* (+)	0.4	< 2.00e-16
	Adjusted R^2	53%		Adjusted R^2	43%	
	P value	1.86E-10		*P* value	< 2.20e-16	
Cross 7	Intercept	3.35	<2.00e-16	Intercept	2.38	< 2.00e-16
	GW5 (B)	-0.42	<2.00e-16	*GW5* (+)	0.4	< 2.00e-16
	Adjusted R^2	93%		Adjusted R^2	43%	
	P value	< 2.20e-16		*P* value	< 2.20e-16	

'B' and '+' in parentheses of the 'Variable' column indicate the low-value genotypes of related genes in F_2GPs and high-value genotypes of related genes in the mini-core collection, respectively.

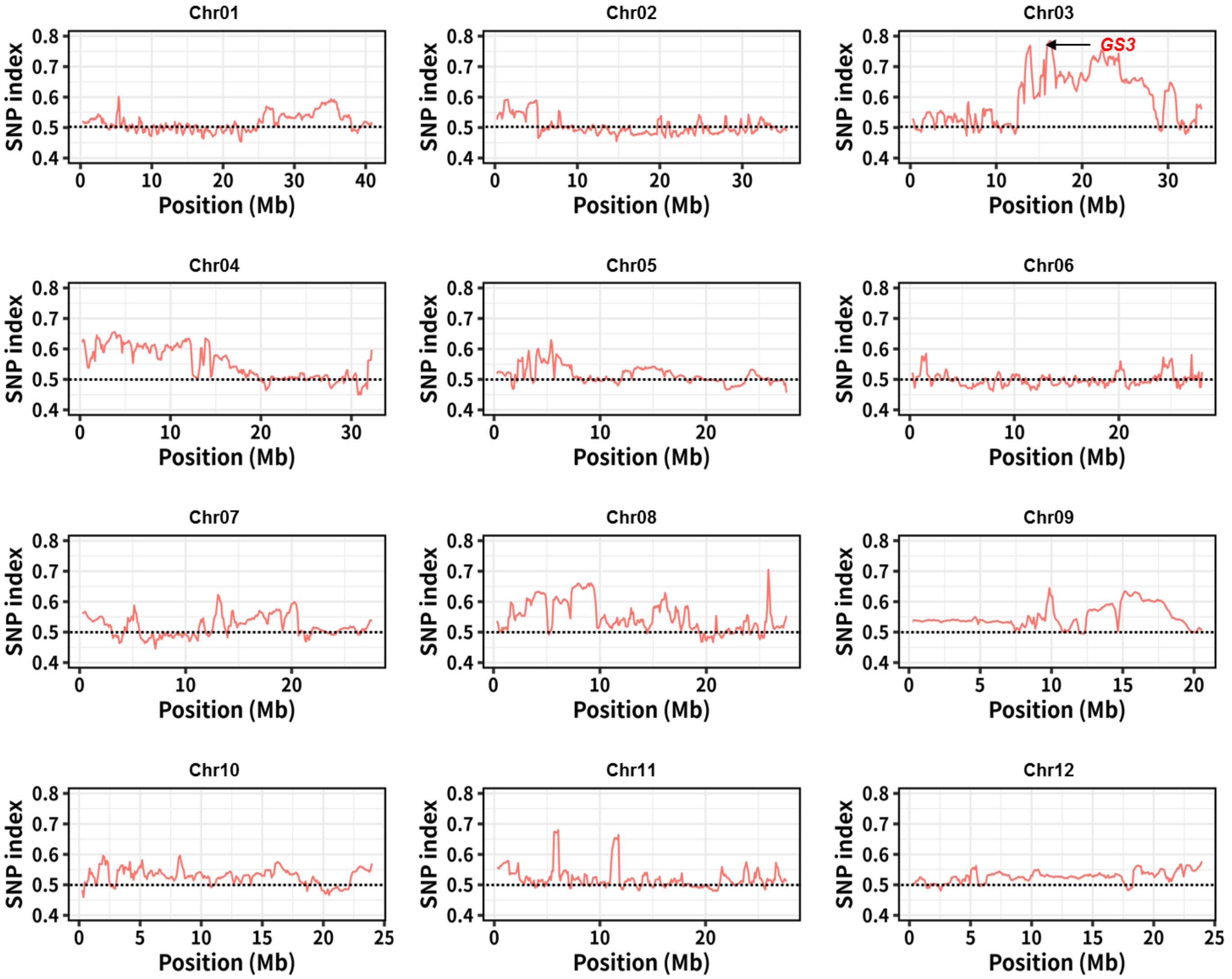

Supplementary Fig. 1. QTL mapping of *GS3* by genome sequencing of two DNA pools. The signal of SNP index was calculated by a 500-kb sliding window and a 100-kb step. The black arrow indicates the signal of *GS3* locus. The dotted lines indicate the SNP index of 0.5.

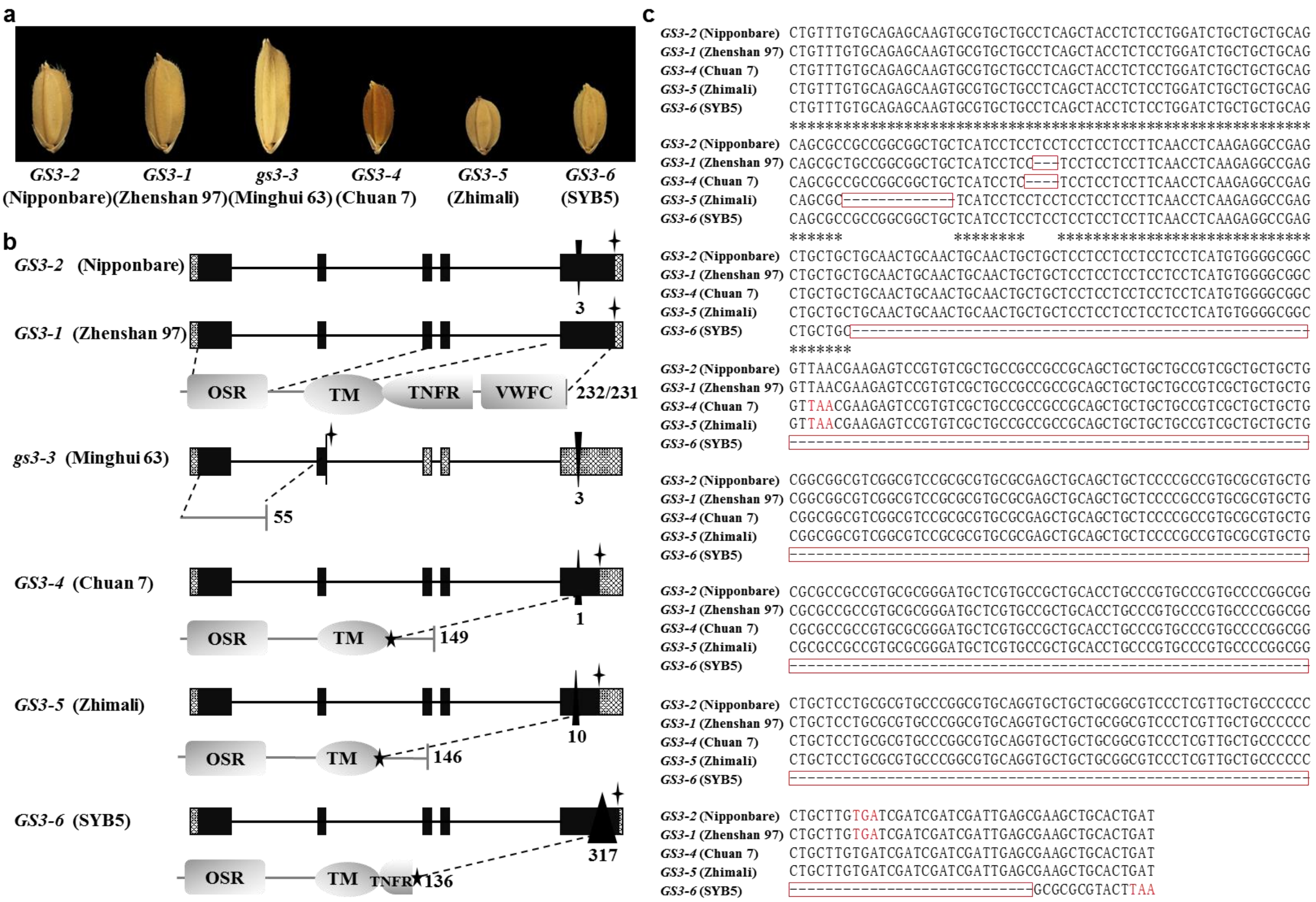

Supplementary Fig. 2. Two new alleles of *GS3* (*GS3-5* and *GS3-6*) discovered by RapMap. a Grain length phenotype of the parents for the grain-length gradient populations with different alleles of the *GS3* gene. **b** Comparison of the gene and protein sequences of six different alleles of *GS3*. Gene structure: hatched boxes represent 5'UTR and 3'UTR, and five exons are represented by black boxes. The thin lines among black boxes represent introns. Black triangles represent a 3-bp insertion (Nipponbare), a 1-bp deletion (Chuan 7), a 10-bp deletion (Zhimali) and a 317-bp deletion (SYB5), compared with that from Zhenshan 97. Stop codon is marked by a four-pointed star. Protein structure (underneath each gene fragment): the domains encoded by Nipponbare or Zhenshan 97 allele are shown. The numbers indicate the the number of amino acids encoding the proteins. The stars indicate the location of protein sequences corresponding to different allelic mutations. **c** Sequence alignment of the fifth exon of different alleles of the functional *GS3*. The red boxes indicate the sites of deletion, and the red characters indicate the stop codon.

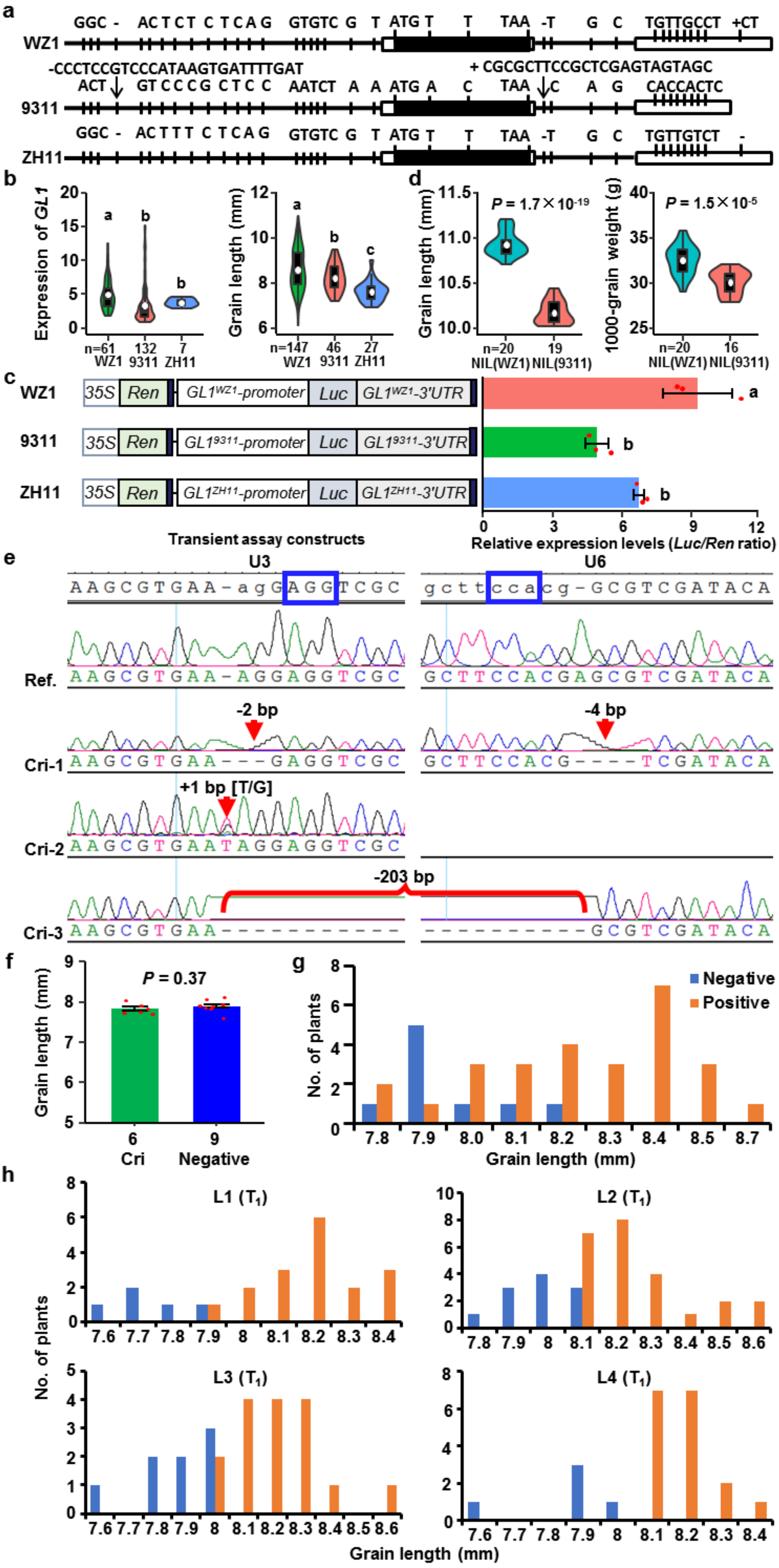
a
WZ1
9311
ZH11
b
Expression of GL1
Grain length (mm)
n=61 WZ1
132 9311
7 ZH11
n=147 WZ1
46 9311
27 ZH11
d
P = 1.7×10-19
P = 1.5×10-5
1000-grain weight (g)
NIL(WZ1) NIL(9311)
c
35S Ren GL1WZ1-promoter Luc GL1WZ1-3'UTR
35S Ren GL19311-promoter Luc GL19311-3'UTR
35S Ren GL1ZH11-promoter Luc GL1ZH11-3'UTR
Transient assay constructs
Relative expression levels (Luc/Ren ratio)
e
U3
U6
Ref.
Cri-1
Cri-2
Cri-3
-2 bp
-4 bp
+1 bp [T/G]
-203 bp
f
P = 0.37
6 Cri
9 Negative
g
Negative
Positive
No. of plants
Grain length (mm)
h
L1 (T1)
L2 (T1)
L3 (T1)
L4 (T1)

Supplementary Fig. 3. Natural variations and function validation of *GL1* by CRISPR and complementation transformation. a Gene structure and natural variations of *GL1*. Black boxes represent exons, and white boxes represent the untranslated regions. The first thin lines represent promoters and the second represents introns. **b** Expression levels and grain-length phenotypes of the three *GL1* haplotypes in the rice mini-core collection based on variations in their promoters and 3'UTR regions. 1-mm young panicles were used for expression analysis. Data are presented as violin plot embedded with box plot which displayed the density distribution (violin), the minima (bottom), maxima (top), center (line in the middle of box), mean (white circle), bounds of the box and whiskers (vertical line). Letters indicated the difference significance adjusted by multiple comparison. n represents the biological independent samples. **c** Transient expression assays on the effects of the three promoters and 3'UTR regions of *GL1* presented in (**a**) on gene expression. Left, constructs with the three promoters and 3'UTR regions from WZ1, 9311 and ZH11. The construct backbone consists of both the target promoter and 3'UTR region controlling the expression of the firefly luciferase reporter gene (*Luc*) and a control 35S promoter regulating the expression of the renilla luciferase reporter gene (*Ren*). Right, corresponding expression levels of Luc relative to Ren. Data are represented mean values +/- SEM. n=3 shows the biologically independent samples. Each data point was plotted on the bars (red dots). Letters above the bars show the statistical significance of multiple comparison. **d** Grain length and weight of the two NILs of NIL(WZ1) and NIL(9311) in the 9311 background. Data are presented as violin plot embedded with box plot which displayed the density distribution (violin), the minima (bottom), maxima (top), center (line in the middle of box), mean (white circle), bounds of the box and whiskers (vertical line). *P* value is generated by two-sided student's *t*-test. n represents the biological independent samples. **e** The genotype of Ref. (ZH11), Cri-1, Cri-2 and Cri-3 at U3 and/or U6 target positions for *GL1*. Blue boxes are PAM sites of their targets. **f** Grain length of homozygous positive and negative CRISPR lines at the U3 and/or the U6 target positions of *GL1* in ZH11. Data are presented as mean +/- SEM. n shows the biologically independent samples. The significance was determined by two-tailed student's *t*-test. **g** Phenotype distribution of all the independent complementation transformation lines for *GL1* in ZH11. **h** Co-segregation test of the four independent complementary transformation lines for *GL1* in T_1 progenies. Source data underlying Supplementary Figure 3c and 3f are provided as a Source Data file.

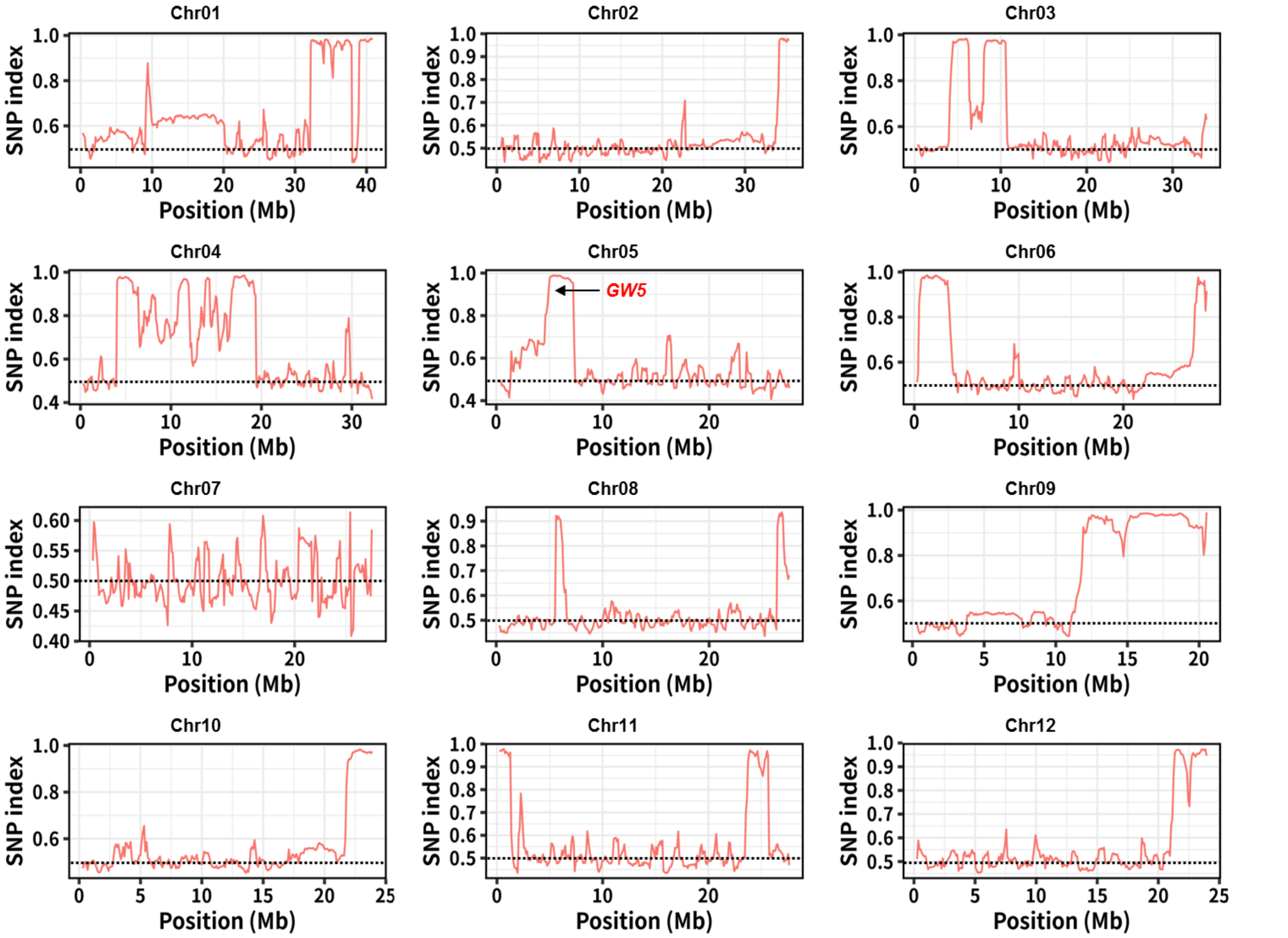

Supplementary Fig. 4. QTL mapping of *GW5* by genome sequencing of two DNA pools. The signal of SNP index was calculated by a 500-kb sliding window and a 100-kb step. The black arrow indicates the signal of *GS3* locus. The dotted lines indicate the SNP index of 0.5.

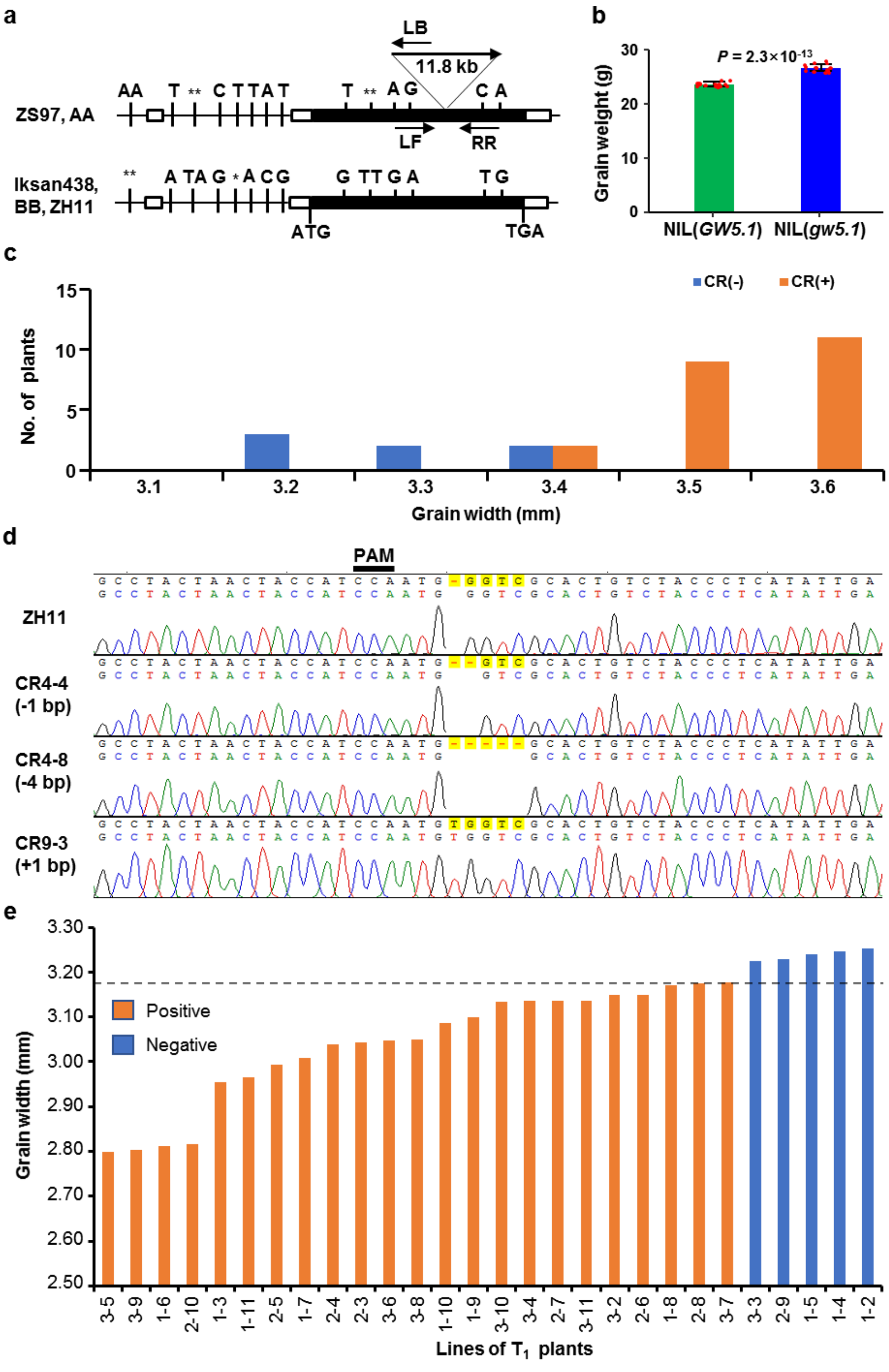

Supplementary Fig. 5. Natural variations and function validation of *GW5.1* by the CRISPR transformation. a Comparative sequencing of ORF1 between two alleles. **b** Comparison of the 1000-grain weight of NIL(*GW5.1*) and NIL(*gw5.1*). Data represent mean +/- SEM (n=15). n represents the number of biologically independent replications. Each data point was plotted on the bars (red dots). Two-sided student's *t*-tests was used to generate *P* values. **c** Distribution of grain width in the independent individuals of edited CR(+) and unedited CR(-) transformants for *GW5.1*. **d** Sequencing analyses of homozygous edited transformants for *GW5.1*. **e** The co-segregation test of T_1 progenies from the T_0 complementation positive plant. Source data underlying Supplementary Figure 5b, 5c, and 5e are provided as a Source Data file.

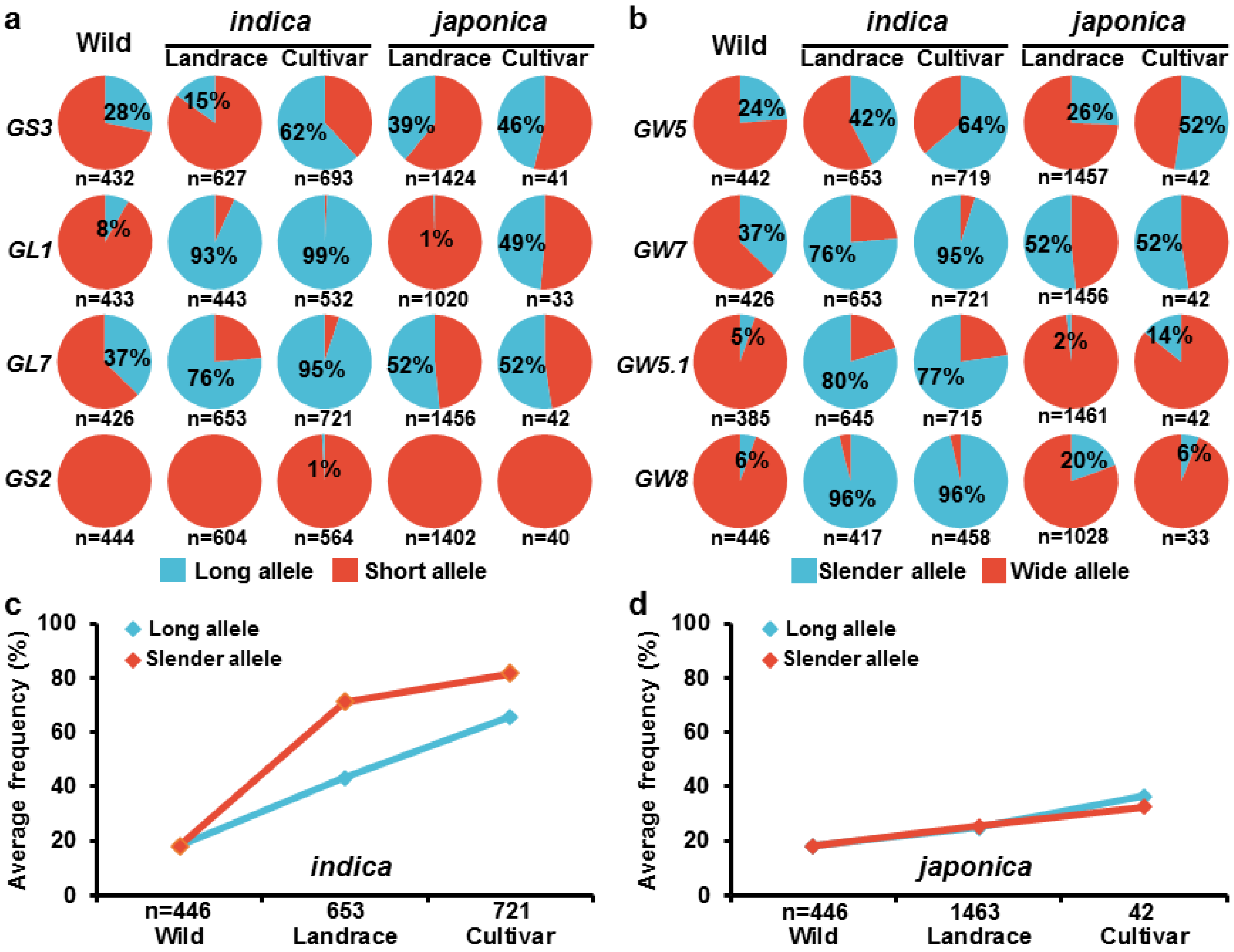

Supplementary Fig. 6. Allele frequencies of grain-length and grain-width genes in *indica* and *japonica* subspecies during the improvement process. **a, b** Frequencies of grain-length (**a**) and grain-width (**b**) alleles in *indica* and *japonica* subspecies. **c, d** The average allele frequencies of long-grain and slender-grain alleles from wild to landrace and from landrace to cultivar in *indica* (**c**) and *japonica* (**d**) subspecies. n represents the biologically independent samples. Source data underlying Supplementary Figure 6c and 6d are provided as a Source Data file.

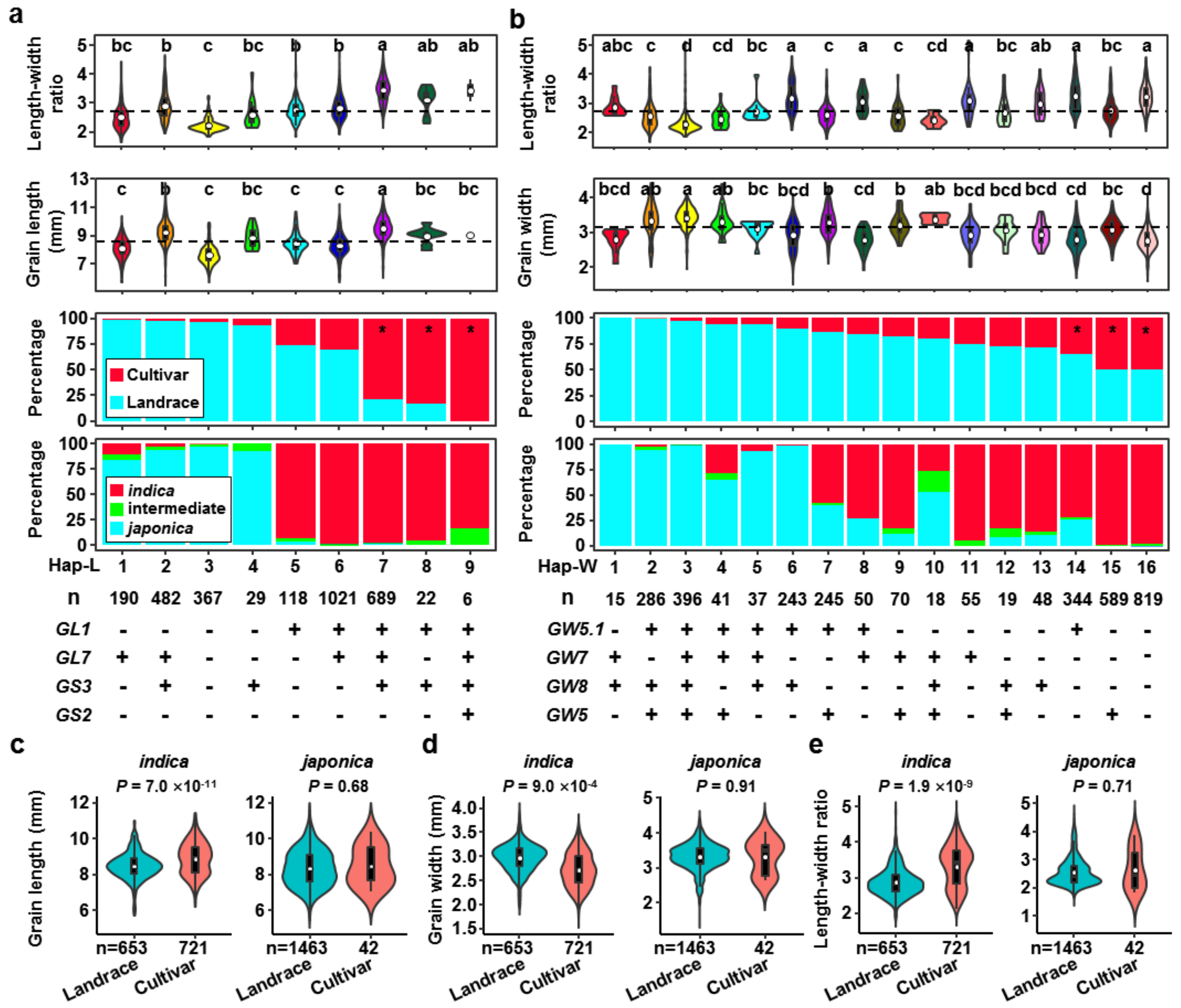

Supplementary Fig. 7. Phenotype effects of different haplotypes of four grain-length genes (a) and four grain-width genes (b), and comparisons of grain length (c), grain width (d) and length-width ratio (e) in landrace and cultivar grouped by *indica* and *japonica* subspecies. Data are presented as violin plot embedded with box plot which displayed the density distribution (violin), the minima (bottom), maxima (top), center (line in the middle of box), mean (white circle), bounds of the box and whiskers (vertical line) (upper panels of **a**-**b**, **c**-**e**). Letters in upper panels of (**a**-**b**) represent the significance of Fisher's least significant difference after analysis of variance. The dotted lines showed the average grain length (**a**), the grain width (**b**) and the length-width ratio (**a**-**b**) of landrace rice. Asterisks mark the three haplotypes with most frequency of cultivars. Hap-L and Hap-W represent haplotypes for grain length (lower panel of **a**) and grain width (lower panel of **b**), respectively. "+" and "-" represent the positive and negative effects of each gene on grain length (**a**) or grain width (**b**), respectively. n shows the biologically independent sample number of each haplotype. *P* values are generated by two-sided Student's *t*-test (**c**–**e**). n represents the number of biologically independent samples (**c**–**e**). Source data are provided as a Source Data file.

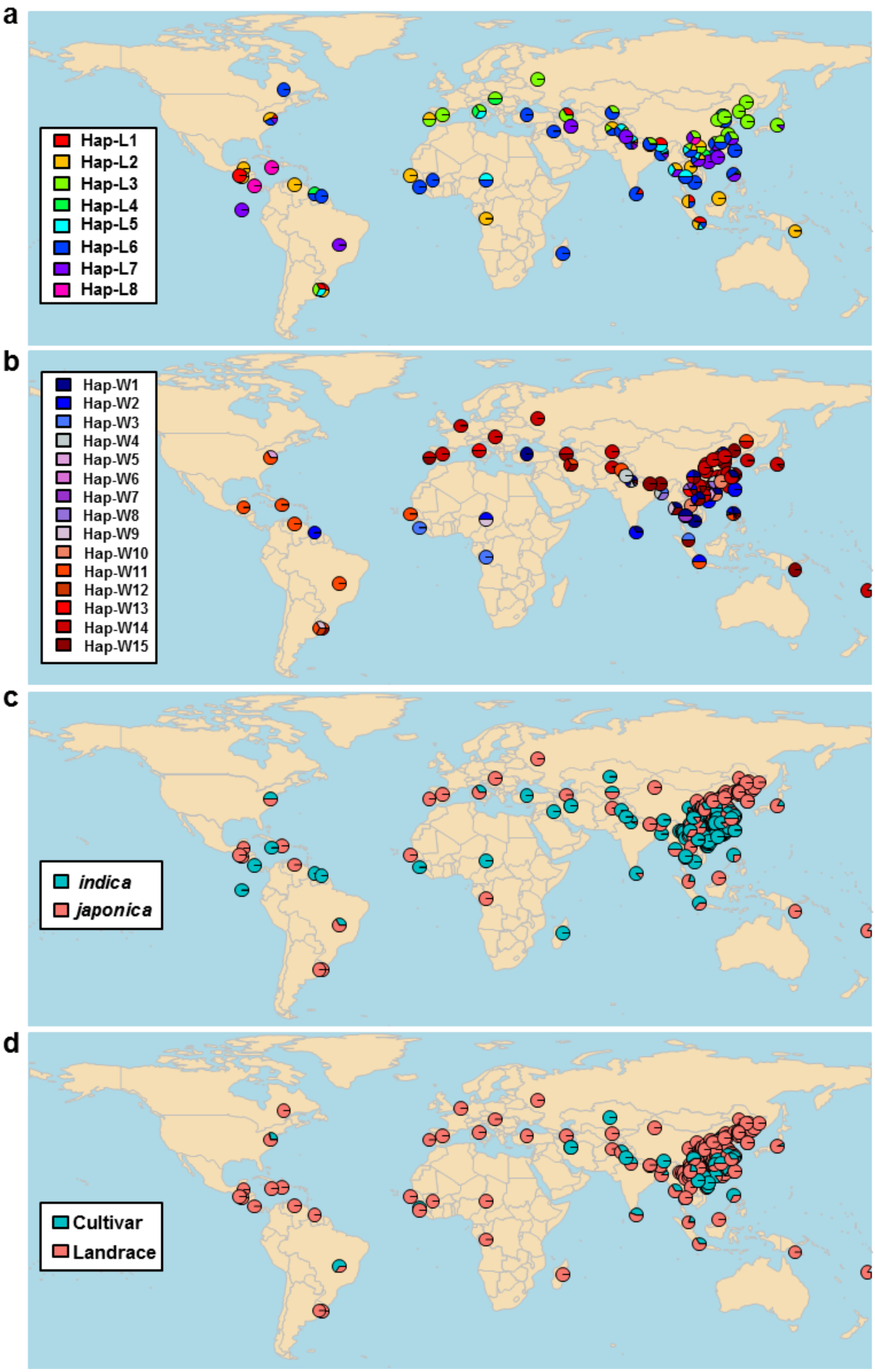

Supplementary Fig. 8. Geographical distribution of the accessions worldwide used for the selection and evolution analyses of the eight grain-size genes. a, b Distributions of the grain-length and grain-width haplotypes in Supplementary Figure 7. Hap-L and Hap-W represent grain-length and grain-width haplotypes in Supplementary Figure 7, respectively. **c** Distribution of the *indica* and *japonica* subspecies. **d** Distribution of the landraces and cultivars. Data were drawn on the map by the *rworldmap* package.

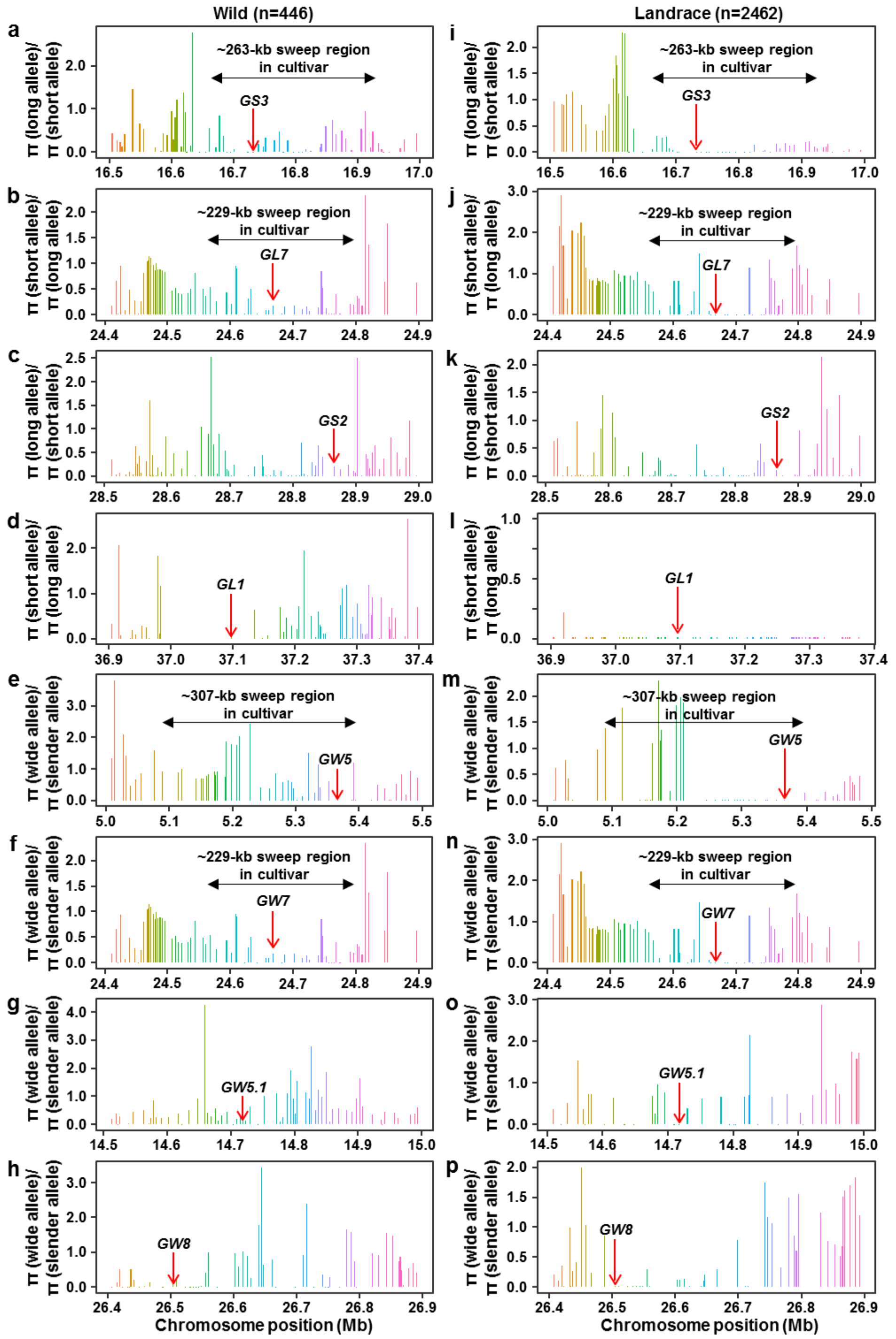

Supplementary Fig. 9. Selection sweeps of the eight grain-size gene loci in wild and landrace rice. a–h Selection sweep scanning across *O. sativa* genome regions of the eight grain-size gene loci in wild rice of 446 accessions. **i–p** Selection sweep scanning across *O. sativa* genome regions of the eight grain-size gene loci in landrace rice of 2462 accessions. Bars indicate the ratios of silent-site nucleotide diversities of each gene in these loci between long- and short-grain allele accessions or wide- and slender-grain allele accessions. The smallest bars are equivalent to a ratio of 0. Red arrows are indicative of each grain-size gene. To compare the differences, the sweep regions in cultivars identified in **Fig. 5a–h** are displayed on each locus.

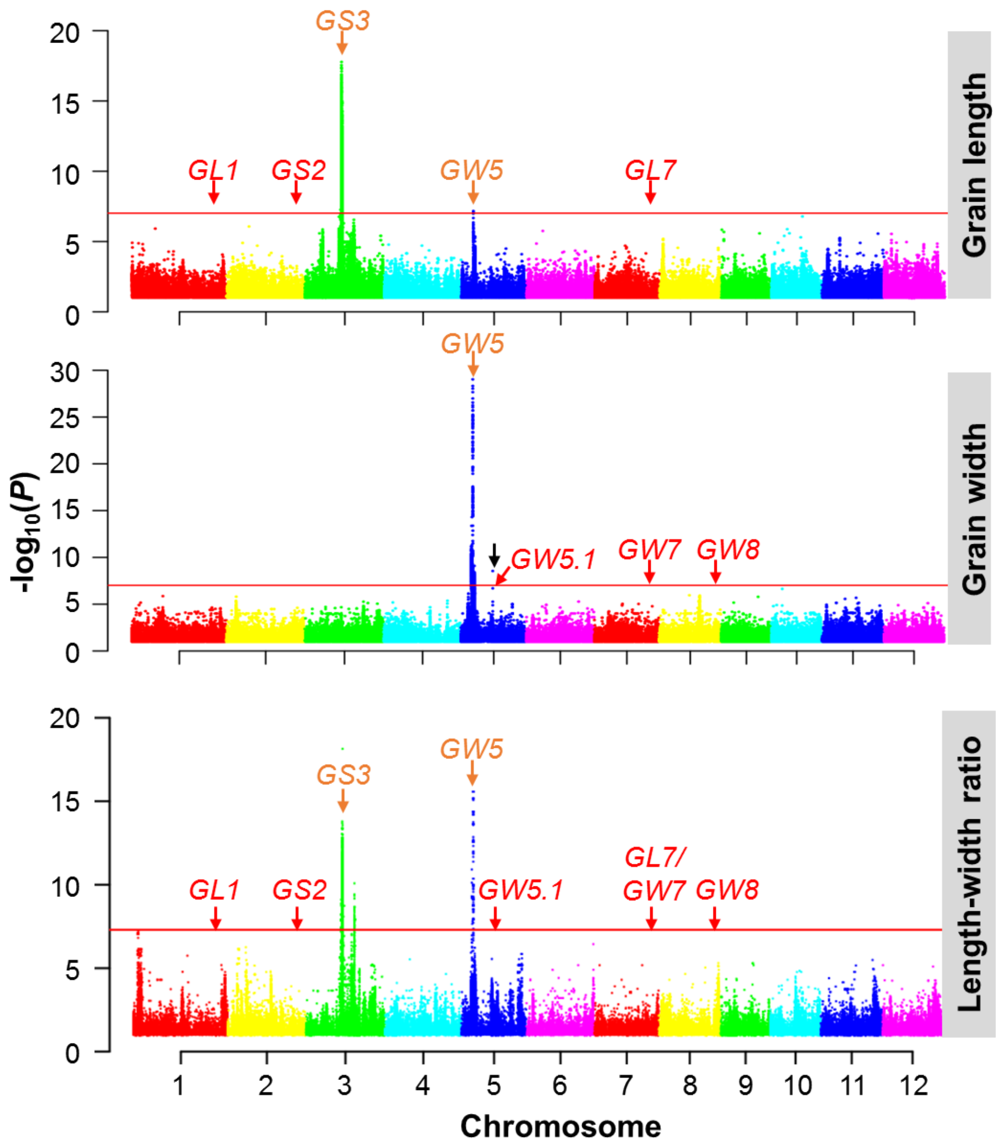

Supplementary Fig. 10. QTLs for grain length, grain width and grain shape (length-width ratio) of rice identified by GWAS using 541 accessions. Red, black and orange arrows indicate the QTL identified by RapMap, GWAS and both, respectively. *GW5.1* is different from the locus indicated by the black arrow.
