# Supplementary material for: The identification of grain size genes by RapMap reveals directional selection during rice domestication
Source: Nat Commun. 2021 Sep 28;12:5673. doi: 10.1038/s41467-021-25961-1 (PMC8478914; doi:10.1038/s41467-021-25961-1)
Supplement: Supplementary file 6 — Description of additional supplementary files [file 41467_2021_25961_MOESM6_ESM.pdf]

Description of additional supplementary files

Title: Supplementary Data 1

Description: Three yield-related agronomic phenotypes of the rice mini-core collection with 541 diverse accessions across the world and genotypes of eight genes for grain length and width identified by RapMap.

Title: Supplementary Data 2

Description: Primer sets used for QTL confirmation, fine mapping and functional analyses.

Title: Supplementary Data 3

Description: Genotypes of the eight grain-size genes in wild rice of 446 accessions.

Title: Supplementary Data 4

Description: Genotypes of the eight grain-size genes and grain size phenotypes in the landraces and cultivars of 2462 and 784 accessions, respectively.
